# Supplementary material for: Testing for saturation in qualitative evidence syntheses: An update of HIV adherence in Africa
Source: PLoS One. 2021 Oct 19;16(10):e0258352. doi: 10.1371/journal.pone.0258352 (PMC8525762; doi:10.1371/journal.pone.0258352)
Supplement: S1 Table — (DOCX) [file pone.0258352.s001.docx]

# S1 Table: Table of included rich studies

| **Study ID** | **Country** | **Participants (n)** | **Phenomenon of interest** | **Data collection methods** |
| --- | --- | --- | --- | --- |
| ***General adults (PLHIV)*** | | | | |
| Bonnington 2017 [1] | Malawi, Uganda, Tanzania, Kenya, Zimbabwe, South Africa | People living with HIV (n=264) and family members of deceased PLHIV (n=48); health care providers (n=54) | Manifestation of stigma at different stages of HIV care continuum and how it influences care and treatment experiences | Repeated In-depth interviews |
| Burman 2019 [2] | South Africa | HIV positive traditionalists on ART (n=30) | Influence of traditionalism on ART adherence | Semi-structured homogenous group discussions |
| Burns 2019 [3] | Kenya, Malawi, Mozambique | PLHIV on second- or third-line ART (n=43); health workers (n=15) | Patients lived experiences of treatment failure and the transition to second (third) line ART | Repeated in-depth interviews, observations |
| Czaicki 2017 [4, 5] | Tanzania | HIV positive patients who recently initiated ART, food insecure who received cash or food incentives (n=29) | The influence of incentives on retention in care and ART adherence | Semi-structured, in-depth interviews |
| Earnshaw 2018 [6] | South Africa | People living with HIV not on ART (n=20); health care providers (n=10) | Barriers to ART initiation in high prevalence settings | Semi-structured interviews |
| Garoon 2018 [7] | Zambia | PLHIV on ART (n=60) | The effects of ART on the social worlds of people living with HIV | In-depth interviews (repeated), participant observations (ethnography) |
| Harris 2016 [8] | South Africa | Patients accessing healthcare services for ART, TB and maternal care (n=45), healthcare providers (n=63) | Access to ART, TB and maternal healthcare in post-apartheid SA | In-depth interviews, observations |
| Horter 2019 [9] | Eswatini | PLHIV (n=30), HCWs (n=20) | The role of stigma on PLHIV experiences with HIV, and engaging with HIV treatment and care services under Treat-all | Repeated in-depth interviews |
| Hurley 2018 [10] | Mali | PLHIV (n=71), HCWs (n=17) | The influence of patient-provider communication on barriers to engagement and reengagement in care | Semi-structured in-depth interviews and focus group discussions |
| Knight 2019 [11] | Kenya | HIV care providers including clinical officers, nurses, HIV counselors, and peripheral support providers such as nutritionists, social workers, and psychosocial/outreach workers (n=60) | The role of gender in the patient–provider relationship and retention in HIV care | In-depth interviews |
| Madhombiro 2018 [12] | Zimbabwe | PLHIV (n=39) | Perceptions and impact of alcohol use | Focus group discussions |
| Moshabela 2017 [13] | Kenya, Uganda, Tanzania, Malawi, Zimbabwe and South Africa | PLHIV (pre-ART, ART and lost-to-follow-up) (n=258), family members of deceased HIV+ people (n=48) and HCWs (n=53) | Manifestations of medical pluralism throughout the HIV care continuum | In-depth interviews |
| Mulqueeny 2019 [14] | South Africa | PLHIV on ART (n=12) | Needs assessment of PLHIV on ART | In-depth interviews and observations |
| Mwamba 2018 [15] | Zambia | PLHIV (currently in care; disengaged from care; in HIV care after transfer to a different clinic; next of kin for deceased patients) (n=69) and HCWs (n=158) | Influence of healthcare delivery on engagement and disengagement from long-term HIV care and treatment. | In-depth interviews and focus group discussions; observations of healthcare facilities' operations |
| Nalugya 2018 [16] | Uganda | HIV positive parents on ART for more than a year (n=38) | Children’s role in supporting HIV-positive parents to self-manage life on ART | Unstructured interviews and semi-structured, in-depth interviews with all participants |
| Nanfuka 2018 [17] | Uganda | PLHIV (n=50) | The role of social capital to overcome risk and sustain adherence to treatment | Semi-structured and narrative interviews, extended case method and participant observation. |
| Ntela 2018 [18] | Democratic Republic of Congo | PLHIV on ART (n=50) | The influence of the media, religious practices and traditional beliefs on compliance to ART | Semi-structured, in-depth interviews |
| Ondenge 2017 [19] | Kenya, Tanzania, Zimbabwe, Malawi, South Africa | PLHIV, family members of recently deceased PLHIV, and HCWs (n=278) | Contextual factors and dynamics that shape patient–provider interactions | Semi-structured, in-depth interviews |
| Patterson 2016 [20] | Zambia | PLHIV, clinic staff, donors (n=25) | Reasons for therapeutic pacifism | In-depth interviews and focus group discussions; participant observation |
| Renju 2017 [21] | Kenya, Tanzania, Zimbabwe, Malawi, South Africa, Uganda | PLHIV initiated on ART (n=168) and HCWs (n=53) | Bodily and relational experience of taking ART and the subsequent effect on retention in HIV care | Semi-structured, in-depth interviews |
| Siril 2017 [22] | Tanzania | PLHIV in care for at least 6 months (n=78) | Building hope and overcoming hopelessness after their HIV diagnosis | Semi-structured, in-depth interviews and focus group discussions |
| Skovdal 2018 [23] | Zimbabwe | PLHIV (n=59) and family members of people who had died from AIDS-related illnesses (n=6) | Intersection of parental obligations of care with HIV treatment-seeking behaviours and retention | Semi-structured, in-depth interviews |
| Skovdal 2017 [24] | Kenya, Tanzania, Malawi, Uganda, Zimbabwe, South Africa | PLHIV (n=225), family members of recently deceased PLHIV (n=48), and HCWs (n=53) | Engagements with HIV services in every-day lives of PLHIV | Semi-structured, in-depth interviews |
| Stern 2017 [25] | South Africa | PLHIV (n=20) who defaulted on ART and then admitted to hospital due to clinical complications and HCWs (n=9) | Barriers to ART adherence and retention in care among patients with various trajectories | Semi-structured, in-depth interviews |
| Topp 2018 [26] | Zambia | PLHIV engaged and disengaged from care (n=55); family members of deceased patients (n=14), HCWs | The role of social and service-related factors influencing retention in HIV care | Semi-structured, in-depth interviews and focus group discussions; participant observation |
| Wachira 2018 [27] | Kenya | PLHIV on ART (n=86) | Perceptions, experiences and expectations of their engagement in care | In-depth interviews and focus group discussions |
| Wamoyi 2017 [28] | Tanzania, Malawi, South Africa | PLHIV at different stages of HIV care (n=107) | Interplay between couple dynamics and the engagement with HIV care and treatment services | Semi-structured, in-depth interviews |
| Weintraub 2018 [29] | South Africa | Recently diagnosed HIV positive people and those undergoing testing (n=24) | Perspectives on Traditional Medicine both in general and in relation to HIV | Semi-structured, in-depth interviews |
| Wringe 2017 [30] | Tanzania, Malawi, South Africa, Zimbabwe, Uganda, Kenya | PLHIV (n=264) and HCWs | Influence of experience of HIV testing on engagement in HIV care among people diagnosed with HIV | Semi-structured, in-depth interviews |
| Zuma 2017 [31] | South Africa | Traditional Health Practitioners (THPs) (n=9) | Traditional Health Practitioners' perceptions and understandings of HIV/AIDS, and their approaches and practices in relation to illnesses of PLHIV | Focus group discussions, community walk (observation) and photovoice techniques |
| Zuma 2018 [32] | South Africa | HIV positive and negative people living in communities (n=52) | Factors influencing choice of sources of healing | Repeat group discussions, repeat semi-structured individual interviews, community walks, photo-voice techniques and participant observation |
| ***Adolescents/children living with HIV*** | | | | |
| Ahsaba 2019 [33] | Uganda | HIV positive adolescents (n=10) and adults, caregivers (n=30) | Mental health and adversities experienced by adolescents with HIV | Focus group discussions and in-depth interviews |
| Bernays 2017 [34] | UK, Ireland, US and Uganda | HIV positive youth aged 10-24 years (n=26) | Experiences with ART | Repeated in-depth interviews; participant observations |
| Enane 2019 [35] | Kenya | Perinatally infected adolescents living with HIV (10-19 years), disclosed and non-disclosed (n=116) and caregivers of ALHIV | Barriers and facilitators to retention in care | Key informant interviews and focus group discussions |
| Kimera 2019 [36] | Uganda | Adolescent peer educators (12-19 years) (n=59) patron teachers of PE's (n=8) | Experiences and challenges of living with HIV in school communities | In-depth interviews and focus group discussions |
| Mackworth-Young 2017 [37] | Zambia | Adolescent girls with HIV (15-19) (n=24) | Experiences of adolescent girls growing up with HIV | Participatory workshops and in-depth interviews |
| McHenry 2017 [38] | Kenya | ALHIV (10 to 15 years) and caregivers of HIV infected children | Experiences and perspectives on stigma | Focus group discussions |
| Mesic 2019 [39] | Zambia | ALHIV (17 to 19) (n=47) | Factors influencing ART initiation, ART adherence, and retention in care | In-depth interviews and focus group discussions |
| Murray 2017 [40] | Uganda | Caregivers of young HIV infected children (n=20) | Relationships between caregiver mental health and HIV-infected child well-being | Semi-structured, in-depth interviews |
| Mutumba 2019 [41] | Uganda | Adolescents living with HIV (12 to 19 years) enrolled in care (n=34) , caregivers (n=34) and HCWs (n=8) | Perceptions, barriers and facilitators of adolescent PLWH self-management role | In-depth interviews and focus group discussions |
| Rosenbaum 2017 [42] | South Africa | Adolescents living with HIV (n=7), their caregivers (n=6) and mental health providers (n=3) | Factors that influence coping with adversities and contributing to well-being and resilience | Photovoice and group discussions, semi-structured interviews |
| Rubincam 2017 [43] | South Africa | Young Adults (HIV status unknown) (n=47) | Alternative explanations, questions, doubts, and skepticism toward official claims about HIV | Focus group discussions |
| Sikstrom 2016 [44] | Malawi | HIV positive children (n=35) and their caregivers, therapy management groups caring for HIV positive children (n=33) , key informants (religious leaders, traditional healers, tobacco estate owners, grandparents, health care workers, HIV programme coordinators) | Barriers to accessing HIV treatment services | Participant observation, longitudinal, semi-structured interviews |
| Woollett 2017 [45] | South Africa | perinatally infected HIV positive adolescents on ART (n=25) | Perceptions of health status, disclosure and mental health | Semi-structured, in-depth interviews |
| Zanoni 2019 [46] | South Africa | Adolescents living with HIV (13 to 24 years) (n=28) and caregivers (n=14) | Facilitators and barriers to retention in care for | Semi-structured, in-depth interviews |
| ***Men*** | | | | |
| Adams 2017 [47] | Eswatini | HIV positive and negative men (n=76) | Test and treat | Informal interviews, participant observation, focus group discussions, in-depth interviews |
| Hendrickson 2019_b [48] | Cote d'Ivoire | Men living with HIV on ART and not on ART, men with unknown HIV status (n=227) | Perceptions of how ART mitigates HIV’s threats to men’s sexuality, economic success, family roles, social status, and health | In-depth interviews, focus group discussions |
| Naugle 2019 [49] | Cote d'Ivoire | Men (25 to 49 years) with 1) unknown HIV status, 2) living with HIV on treatment, 3) MLHIV not on treatment (n=227) | The role of masculinity in men’s engagement in the HIV care continuum | In-depth interviews and focus group discussions |
| Russell 2019 [50] | Uganda | Men living with HIV on ART (n=18) | Influence of ART on men’s refashioning of their masculine identities | Repeat semi-structured, in-depth interviews |
| Sileo 2019_a [51] | Uganda | HIV infected male fisherfolk on ART (n=30) | Alcohol consumption and ART adherence | Semi-structured, in-depth interviews |
| Sileo 2019_b [52] | Uganda | HIV infected male fisherfolk on ART (n=30) | The role of masculinity on men’s engagement in HIV care | Semi-structured, in-depth interviews |
| Tibbels 2019 [53] | Cote d'Ivoire | Men living with HIV and those with unknown HIV status (n=227) | Perceptions on accessing care | In-depth interviews and focus group discussions |
| Tocco 2017 [54] | Nigeria | HIV positive Muslim men (n=30), key informants (n=30) (biomedical practitioners, drug adherence officers and home-based care volunteers, Islamic prophetic healers, leaders of Muslim organizations, and government health officials), members of the HIV support group | Balancing ART with religious tenets and obligations | Participant observation; semi-structured, in-depth interviews |
| ***Women*** | | | | |
| Dlamini-Simelane 2017 [55] | Eswatini | Women with HIV (n=2) | Factors influencing access to treatment | In-depth interviews and participant observation |
| Hussen 2019 [56] | Ethiopia | female HIV positive peer educators (n=19) | HIV-positive women’s partnerships over the life course and implications for engaging in care | Semi-structured in-depth interviews |
| ***Pregnant/postpartum women*** | | | | |
| Fords 2017 [57] | South Africa | HIV positive pregnant women on ART (n=10) | Lived experiences of women diagnosed with HIV for the first time during the antenatal period | Semi-structured, in-depth interviews |
| Gill 2017 [58] | Rwanda | HIV-positive postpartum women (n=112) | Attitudes and norms affecting women’s postpartum ART adherence | Semi-structured, in-depth interviews |
| Heerink 2019 [59] | South Africa | HIV positive and negative women in the postpartum period (n=31), HCWs in the antenatal clinic or postnatal ward (n=14) | Attitudes of HCWs in the context of HIV and PMTCT related care | In-depth, semi-structured interviews and focus group discussions |
| Katirayi 2017 [60] | Malawi and Zimbabwe | Pregnant and postpartum women initiated on Option B+ (n=298) and HCWs (n=68) | Challenges of HIV testing and treatment and how men affect their partner’s ability to initiate and adhere to ART. | In-depth interviews and focus group discussions |
| Marais 2019 [61] | South Africa | HIV-positive pregnant women with recent histories of intimate partner violence (IPV) (n=12) | Experiences of recent IPV in HIV positive pregnant women | Semi-structured in-depth interviews |
| McLean 2017 [62] | Malawi, Tanzania, Uganda, | HIV positive women who were currently pregnant or had been since Option B+ (n=22); HCWs (n=15) | Factors influencing acceptance and adherence to Option B+ | Semi-structured, in-depth interviews |
| Sakyi 2019 [63] | Ghana | Postpartum women living with ART (n=30) | Influence of caring for low-birth-weight infants on maternal ART adherence and retention in care. | Semi-structured, in-depth interviews |
| Spangler 2018 [64] | Kenya | HIV positive pregnant or postpartum women (n=38) and their male partners (aware of status) | Perceptions of HIV stigma and self-disclosure, and their effects on engagement in prevention of mother-to-child transmission and HIV care | Semi-structured, in-depth interviews |
| Watt 2018 [65] | South Africa | HIV positive pregnant women (n=20) | HIV disclosure decision-making and processes | Two semi-structured, in-depth interviews (one during pregnancy, one during postpartum period) |
| ***Seroconcordant/sero-discordant couples*** | | | | |
| Conroy 2017_b [66] | South Africa | Couples with at least one HIV-positive partner (n=24 couples) | Primary partners' beliefs and influence on alcohol consumption and ART adherence | Semi-structured, in-depth interviews |
| Conroy 2017_a [67] | South Africa | Couples with at least one HIV-positive partner (n=24 couples) | How relationships interfere with or support adherence | Semi-structured, in-depth interviews |
| Conroy 2018 [68] | Malawi | Married couples of whom at least one partner was HIV positive and eligible for ART, or on ART (n=25 couples) | Influence of relationships and the interplay of marital infidelity, food insecurity, and couple instability on ART adherence | In-depth interviews (separate interviews with husbands and wives) |
| Conroy 2019_a [69] | Malawi | Malawian couples on ART where at least one partner is HIV positive (n=25 couples) | Influence of alcohol consumption and couple relationships and ART adherence | In-depth interviews (repeated interviews for some) |
| Conroy 2019_b [70] | Malawi | Couples with at least one HIV-positive partner and at least one partner who had used alcohol in the past year (n=23 couples) | Barriers and facilitators of alcohol use in couples living with HIV | In-depth interviews (separate for husband and wives) |
| Monroe-Wise 2019 [71] | Kenya | PLHIV in care, their partners and HCWs (n=49) | Opportunities and barriers to assisted partner notification systems | In-depth interviews and focus group discussions |
| Ware 2018 [72] | Uganda | PLHIV and their partners (n=93 couples) | Organization of services and couples’ responses to integrated PrEP and ART | In-depth interviews, field observations |
| ***Men who have sex with men*** | | | | |
| Graham 2018 [73] | Kenya | HIV infected gay, bisexual, and other men who have sex with men (n=30) | Barriers and facilitators of HIV care engagement and ART adherence | In-depth interviews |
| Matovu 2019 [74] | Uganda | HCWs (n=48) | Perceptions, experiences and readiness to provide HIV services to MSM and FSWs | Semi-structured interviews |
| Micheni 2017 [75] | Kenya | HCWs experienced in providing care to HIV positive MSM (n=29) | Factors affecting ART adherence and engagement with care | Focus group discussions |
| ***Alcohol and other drug users*** | | | | |
| Cooke 2017 [76] | Tanzania | Opoid treatment patients (OTP) living with HIV (n=20), OTP healthcare providers (n=12) | Perspectives of implementing an integrated methadone and ART service delivery model at an opioid treatment program | Semi-structured, in-depth interviews |
| Magidson 2019 [77] | South Africa | PLHIV on ART and reported moderate AOD use (n=19); HCWs (n=11) | Barriers and facilitators to integrating services to treat problematic alcohol or other drugs use in HIV care | Semi-structured, in-depth interviews |
| ***Older PLHIV*** | | | | |
| Kiplagat 2018 [78] | Kenya | PLHIV over the age of 50, currently receiving care 9n=57) | Challenges faced by older adults living with HIV in accessing and engaging in HIV care services | In-depth interviews and focus group discussions |
| Schatz 2019 [79] | Uganda | older PLHIV (age 50 to 96) on ART or waiting to initiate ART (n=40) | Barriers and facilitators to accessing and adhering to ART | Semi-structured, in-depth interviews |
| ***People living with disabilities*** | | | | |
| Schenk 2020 [80] | Ghana, Uganda and Zambia | Key informants (government officials and HIV service providers, including national and local disabled persons organisations) (n=21) and disabled persons with and without HIV (n=263) | Barriers and facilitators to access to HIV services for persons with disabilities | Semi-structured interviews and focus group discussions |
| ***Migrants*** | | | | |
| Steenberg 2020 [81] | South Africa | HIV positive Mozambican migrants attending HIV care in Johannesburg, South Africa (n=21) | Social complexities of living with ART and disclosure of serostatus | Ethnographic approach involving informal conversation, interaction and observations, filed notes, semi-structured life history interviews |

## References to studies

1. Bonnington O, Wamoyi J, Ddaaki W, Bukenya D, Ondenge K, Skovdal M, et al. Changing forms of HIV-related stigma along the HIV care and treatment continuum in sub-Saharan Africa: a temporal analysis. Sex Transm Infect. 2017;93(Suppl 3). doi: <https://dx.doi.org/10.1136/sextrans-2016-052975>.

2. Burman C, Aphane M. Improved adherence to anti-retroviral therapy among traditionalists: reflections from rural South Africa. Afr Health Sci. 2019;19(1):1422-32. doi: <https://dx.doi.org/10.4314/ahs.v19i1.15>.

3. Burns R, Borges J, Blasco P, Vandenbulcke A, Mukui I, Magalasi D, et al. 'I saw it as a second chance': A qualitative exploration of experiences of treatment failure and regimen change among people living with HIV on second- and third-line antiretroviral therapy in Kenya, Malawi and Mozambique. Glob Public Health. 2019;14(8):1112-24. doi: <https://dx.doi.org/10.1080/17441692.2018.1561921>.

4. Czaicki NL. Understanding and informing interventions to improve antiretroviral adherence: Three papers on antiretroviral adherence in Sub-Saharan Africa. 2019;80. PubMed PMID: 2018-52508-008.

5. Czaicki NL, Mnyippembe A, Blodgett M, Njau P, McCoy SI. It helps me live, sends my children to school, and feeds me: a qualitative study of how food and cash incentives may improve adherence to treatment and care among adults living with HIV in Tanzania. AIDS Care. 2017;29(7):876-84. doi: <https://dx.doi.org/10.1080/09540121.2017.1287340>.

6. Earnshaw VA, Bogart LM, Courtney I, Zanoni H, Bangsberg DR, Orrell C, et al. Exploring Treatment Needs and Expectations for People Living with HIV in South Africa: A Qualitative Study. AIDS Behav. 2018;22(8):2543-52. doi: 10.1007/s10461-018-2101-x. PubMed PMID: 29619585.

7. Garoon J. "These African stories": Life, labor, and dying in northern Zambia. Soc Sci Med. 2018;201:9-17. doi: <https://dx.doi.org/10.1016/j.socscimed.2018.01.021>.

8. Harris B, Eyles J, Goudge J. Ways of doing: Restorative practices, governmentality, and provider conduct in post-apartheid health care. Med Anthropol. 2016;35(6):572-87. doi: 10.1080/01459740.2016.1173691. PubMed PMID: 2016-54064-011.

9. Horter S, Bernays S, Thabede Z, Dlamini V, Kerschberger B, Pasipamire M, et al. "I don't want them to know": how stigma creates dilemmas for engagement with Treat-all HIV care for people living with HIV in Eswatini. Afr J AIDS Res. 2019;18(1):27-37. doi: <https://dx.doi.org/10.2989/16085906.2018.1552163>.

10. Hurley EA, Harvey SA, Winch PJ, Keita M, Roter DL, Doumbia S, et al. The Role of Patient-Provider Communication in Engagement and Re-engagement in HIV Treatment in Bamako, Mali: A Qualitative Study. J Health Commun. 2018;23(2):129-43. doi: <https://dx.doi.org/10.1080/10810730.2017.1417513>.

11. Knight J, Wachira J, Kafu C, Braitstein P, Wilson IB, Harrison A, et al. The Role of Gender in Patient-Provider Relationships: A Qualitative Analysis of HIV Care Providers in Western Kenya with Implications for Retention in Care. AIDS Behav. 2019;23(2):395-405. doi: <https://dx.doi.org/10.1007/s10461-018-2265-4>.

12. Madhombiro M, Marimbe-Dube B, Dube M, Kaiyo-Utete M, Paradzai A, Chibanda D, et al. Perceptions of alcohol use in the context of HIV treatment: a qualitative study. HIV AIDS (Auckl). 2018;10:47-55. doi: <https://dx.doi.org/10.2147/HIV.S150095>.

13. Moshabela M, Bukenya D, Darong G, Wamoyi J, McLean E, Skovdal M, et al. Traditional healers, faith healers and medical practitioners: the contribution of medical pluralism to bottlenecks along the cascade of care for HIV/AIDS in Eastern and Southern Africa. Sexually Transmitted Infections. 2017;93(Suppl 3). doi: 10.1136/sextrans-2016-052974.

14. Mulqueeny DM, Taylor M. Does the public antiretroviral treatment programme meet patients' needs? A study at four hospitals in eThekwini, KwaZulu-Natal, South Africa. Afr J Prim Health Care Fam Med. 2019;11(1):e1-e11. doi: <https://dx.doi.org/10.4102/phcfm.v11i1.1824>.

15. Mwamba C, Sharma A, Mukamba N, Beres L, Geng E, Holmes CB, et al. 'They care rudely!': resourcing and relational health system factors that influence retention in care for people living with HIV in Zambia. BMJ global health. 2018;3(5):e001007. doi: <https://dx.doi.org/10.1136/bmjgh-2018-001007>.

16. Nalugya R, Russell S, Zalwango F, Seeley J. The role of children in their HIV-positive parents' management of antiretroviral therapy in Uganda. Afr J AIDS Res. 2018;17(1):37-46. doi: <https://dx.doi.org/10.2989/16085906.2017.1394332>.

17. Nanfuka EK, Kyaddondo D, Ssali SN, Asingwire N. Social capital and resilience among people living on antiretroviral therapy in resource-poor Uganda. PLoS One. 2018;13(6):e0197979. Epub 2018/06/12. doi: 10.1371/journal.pone.0197979. PubMed PMID: 29889849; PubMed Central PMCID: PMCPMC5995438.

18. Ntela S-DM, Goutte N, Morvillers J-M, Crozet C, Ahouah M, Omanyondo-Ohambe M-C, et al. Observance to antiretroviral treatment in the rural region of the Democratic Republic of Congo: a cognitive dissonance. Pan Afr Med J. 2018;31:159. doi: <https://dx.doi.org/10.11604/pamj.2018.31.159.15132>.

19. Ondenge K, Renju J, Bonnington O, Moshabela M, Wamoyi J, Nyamukapa C, et al. 'I am treated well if I adhere to my HIV medication': putting patient-provider interactions in context through insights from qualitative research in five sub-Saharan African countries. Sex Transm Infect. 2017;93(Suppl 3). Epub 2017/07/25. doi: 10.1136/sextrans-2016-052973. PubMed PMID: 28736392; PubMed Central PMCID: PMCPMC5739840.

20. Patterson AS. Engaging therapeutic citizenship and clientship: Untangling the reasons for therapeutic pacifism among people living with HIV in urban Zambia. Glob Public Health. 2016;11(9):1121-34. doi: 10.1080/17441692.2015.1070053. PubMed PMID: 117745406. Language: English. Entry Date: 20160909. Revision Date: 20190209. Publication Type: Article.

21. Renju J, Moshabela M, McLean E, Ddaaki W, Skovdal M, Odongo F, et al. 'Side effects' are 'central effects' that challenge retention in HIV treatment programmes in six sub-Saharan African countries: a multicountry qualitative study. Sex Transm Infect. 2017;93(Suppl 3). doi: <https://dx.doi.org/10.1136/sextrans-2016-052971>.

22. Siril H, Fawzi MCS, Todd J, Wyatt M, Kilewo J, Ware N, et al. Hopefulness Fosters Affective and Cognitive Constructs for Actions to Cope and Enhance Quality of Life among People Living with HIV in Dar Es Salaam, Tanzania. J Int Assoc Provid AIDS Care. 2017;16(2):140-8. doi: <https://dx.doi.org/10.1177/2325957414539195>.

23. Skovdal M, Maswera R, Kadzura N, Nyamukapa C, Rhead R, Wringe A, et al. Parental obligations, care and HIV treatment: How care for others motivates self-care in Zimbabwe. J Health Psychol. 2018:1359105318788692. doi: <https://dx.doi.org/10.1177/1359105318788692>.

24. Skovdal M, Wringe A, Seeley J, Renju J, Paparini S, Wamoyi J, et al. Using theories of practice to understand HIV-positive persons varied engagement with HIV services: a qualitative study in six Sub-Saharan African countries. Sex Transm Infect. 2017;93(Suppl 3). Epub 2017/07/25. doi: 10.1136/sextrans-2016-052977. PubMed PMID: 28736396; PubMed Central PMCID: PMCPMC5739842.

25. Stern E, Colvin C, Gxabagxaba N, Schutz C, Burton R, Meintjes G. Conceptions of agency and constraint for HIV-positive patients and healthcare workers to support long-term engagement with antiretroviral therapy care in Khayelitsha, South Africa. Afr J AIDS Res. 2017;16(1):19-29. doi: <https://dx.doi.org/10.2989/16085906.2017.1285795>.

26. Topp SM, Mwamba C, Sharma A, Mukamba N, Beres LK, Geng E, et al. Rethinking retention: Mapping interactions between multiple factors that influence long-term engagement in HIV care. PLoS One. 2018;13(3):e0193641. doi: <https://dx.doi.org/10.1371/journal.pone.0193641>.

27. Wachira J, Genberg B, Kafu C, Braitstein P, Laws MB, Wilson IB. Experiences and expectations of patients living with HIV on their engagement with care in Western Kenya. Patient Prefer Adherence. 2018;12:1393-400. doi: <https://dx.doi.org/10.2147/PPA.S168664>.

28. Wamoyi J, Renju J, Moshabela M, McLean E, Nyato D, Mbata D, et al. Understanding the relationship between couple dynamics and engagement with HIV care services: insights from a qualitative study in Eastern and Southern Africa. Sex Transm Infect. 2017;93(Suppl 3). doi: <https://dx.doi.org/10.1136/sextrans-2016-052976>.

29. Weintraub A, Mantell JE, Holt K, Street RA, Wilkey C, Dawad S, et al. 'These people who dig roots in the forests cannot treat HIV': Women and men in Durban, South Africa, reflect on traditional medicine and antiretroviral drugs. Glob Public Health. 2018;13(1):115-27. doi: 10.1080/17441692.2017.1359326. PubMed PMID: 28793809.

30. Wringe A, Moshabela M, Nyamukapa C, Bukenya D, Ondenge K, Ddaaki W, et al. HIV testing experiences and their implications for patient engagement with HIV care and treatment on the eve of 'test and treat': findings from a multicountry qualitative study. Sex Transm Infect. 2017;93(Suppl 3). doi: <https://dx.doi.org/10.1136/sextrans-2016-052969>.

31. Zuma T, Wight D, Rochat T, Moshabela M. Traditional health practitioners' management of HIV/AIDS in rural South Africa in the era of widespread antiretroviral therapy. Glob Health Action. 2017;10(1):1352210. doi: 10.1080/16549716.2017.1352210. PubMed PMID: 28771116.

32. Zuma T, Wight D, Rochat T, Moshabela M. Navigating Multiple Sources of Healing in the Context of HIV/AIDS and Wide Availability of Antiretroviral Treatment: A Qualitative Study of Community Participants' Perceptions and Experiences in Rural South Africa. Frontiers in public health. 2018;6:73. doi: <https://dx.doi.org/10.3389/fpubh.2018.00073>.

33. Ashaba S, Cooper-Vince CE, Vorechovska D, Rukundo GZ, Maling S, Akena D, et al. Community beliefs, HIV stigma, and depression among adolescents living with HIV in rural Uganda. Afr J AIDS Res. 2019;18(3):169-80. doi: <https://dx.doi.org/10.2989/16085906.2019.1637912>.

34. Bernays S, Paparini S, Seeley J, Rhodes T. "Not Taking it Will Just be Like a Sin": Young People Living with HIV and the Stigmatization of Less-Than-Perfect Adherence to Antiretroviral Therapy. Med Anthropol. 2017;36(5):485-99. doi: <https://dx.doi.org/10.1080/01459740.2017.1306856>.

35. Enane LA, Apondi E, Toromo J, Bosma C, Ngeresa A, Nyandiko W, et al. "A problem shared is half solved" - a qualitative assessment of barriers and facilitators to adolescent retention in HIV care in western Kenya. AIDS Care. 2019:1-9. doi: <https://dx.doi.org/10.1080/09540121.2019.1668530>.

36. Kimera E, Vindevogel S, Rubaihayo J, Reynaert D, De Maeyer J, Engelen A-M, et al. Youth living with HIV/AIDS in secondary schools: perspectives of peer educators and patron teachers in Western Uganda on stressors and supports. Sahara j. 2019;16(1):51-61. doi: <https://dx.doi.org/10.1080/17290376.2019.1626760>.

37. Mackworth-Young CR, Bond V, Wringe A, Konayuma K, Clay S, Chiiya C, et al. "My mother told me that I should not": a qualitative study exploring the restrictions placed on adolescent girls living with HIV in Zambia. Journal of the International AIDS Society. 2017;20(4). doi: <https://dx.doi.org/10.1002/jia2.25035>.

38. McHenry MS, Nyandiko WM, Scanlon ML, Fischer LJ, McAteer CI, Aluoch J, et al. HIV Stigma: Perspectives from Kenyan Child Caregivers and Adolescents Living with HIV. J Int Assoc Provid AIDS Care. 2017;16(3):215-25. doi: <https://dx.doi.org/10.1177/2325957416668995>.

39. Mesic A, Halim N, MacLeod W, Haker C, Mwansa M, Biemba G. Facilitators and Barriers to Adherence to Antiretroviral Therapy and Retention in Care Among Adolescents Living with HIV/AIDS in Zambia: A Mixed Methods Study. AIDS Behav. 2019;23(9):2618-28. doi: <https://dx.doi.org/10.1007/s10461-019-02533-5>.

40. Murray SM, Familiar I, Nakasujja N, Winch PJ, Gallo JJ, Opoka R, et al. Caregiver mental health and HIV-infected child wellness: perspectives from Ugandan caregivers. AIDS Care. 2017;29(6):793-9. doi: <https://dx.doi.org/10.1080/09540121.2016.1263722>.

41. Mutumba M, Musiime V, Mugerwa H, Nakyambadde H, Gautam A, Matama C, et al. Perceptions of HIV Self-Management Roles and Challenges in Adolescents, Caregivers, and Health Care Providers. JANAC: Journal of the Association of Nurses in AIDS Care. 2019;30(4):415-27. doi: 10.1097/JNC.0000000000000011. PubMed PMID: 139218567. Language: English. Entry Date: 20191031. Revision Date: 20191031. Publication Type: Article. Journal Subset: Core Nursing.

42. Rosenbaum L. Exploring the social ecological actors that contribute to the resilience of adolescents living with HIV in South Africa: A photovoice study. 2018;78. PubMed PMID: 2017-43830-104.

43. Rubincam C. "It's natural to look for a source": A qualitative examination of alternative beliefs about HIV and AIDS in Cape Town, South Africa. Public understanding of science (Bristol, England). 2017;26(3):369-84. doi: <https://dx.doi.org/10.1177/0963662515611823>.

44. Sikstrom L. 'He is almost like other children': An ethnography of Malawi's national pediatric HIV treatment programme. 2017;77. PubMed PMID: 2016-47709-036.

45. Woollett N, Black V, Cluver L, Brahmbhatt H. Reticence in disclosure of HIV infection and reasons for bereavement: impact on perinatally infected adolescents' mental health and understanding of HIV treatment and prevention in Johannesburg, South Africa. Afr J AIDS Res. 2017;16(2):175-84. doi: <https://dx.doi.org/10.2989/16085906.2017.1337646>.

46. Zanoni BC, Sibaya T, Cairns C, Haberer JE. Barriers to Retention in Care are Overcome by Adolescent-Friendly Services for Adolescents Living with HIV in South Africa: A Qualitative Analysis. AIDS Behav. 2019;23(4):957-65. doi: <https://dx.doi.org/10.1007/s10461-018-2352-6>.

47. Adams AK, Zamberia AM. "I will take ARVs once my body deteriorates": an analysis of Swazi men's perceptions and acceptability of Test and Start. Afr J AIDS Res. 2017;16(4):295-303. doi: <https://dx.doi.org/10.2989/16085906.2017.1362015>.

48. Hendrickson ZM, Naugle DA, Tibbels N, Dosso A, M Van Lith L, Mallalieu EC, et al. "You Take Medications, You Live Normally": The Role of Antiretroviral Therapy in Mitigating Men's Perceived Threats of HIV in Cote d'Ivoire. AIDS Behav. 2019;23(9):2600-9. doi: <https://dx.doi.org/10.1007/s10461-019-02614-5>.

49. Naugle DA, Tibbels NJ, Hendrickson ZM, Dosso A, Van Lith L, Mallalieu EC, et al. Bringing fear into focus: The intersections of HIV and masculine gender norms in Cote d'Ivoire. PLoS One. 2019;14(10):e0223414. doi: <https://dx.doi.org/10.1371/journal.pone.0223414>.

50. Russell S. Men's Refashioning of Masculine Identities in Uganda and Their Self-Management of HIV Treatment. Qual Health Res. 2019;29(8):1199-212. doi: <https://dx.doi.org/10.1177/1049732318823717>.

51. Sileo KM, Kizito W, Wanyenze RK, Chemusto H, Musoke W, Mukasa B, et al. A qualitative study on alcohol consumption and HIV treatment adherence among men living with HIV in Ugandan fishing communities. AIDS Care. 2019;31(1):35-40. doi: <https://dx.doi.org/10.1080/09540121.2018.1524564>.

52. Sileo KM, Reed E, Kizito W, Wagman JA, Stockman JK, Wanyenze RK, et al. Masculinity and engagement in HIV care among male fisherfolk on HIV treatment in Uganda. Cult Health Sex. 2019;21(7):774-88. doi: <https://dx.doi.org/10.1080/13691058.2018.1516299>.

53. Tibbels NJ, Hendrickson ZM, Naugle DA, Dosso A, Van Lith L, Mallalieu EC, et al. Men's perceptions of HIV care engagement at the facility- and provider-levels: Experiences in Cote d'Ivoire. PLoS One. 2019;14(3):e0211385. doi: <https://dx.doi.org/10.1371/journal.pone.0211385>.

54. Tocco JU. The Islamification of antiretroviral therapy: Reconciling HIV treatment and religion in northern Nigeria. Soc Sci Med. 2017;190:75-82. doi: <https://dx.doi.org/10.1016/j.socscimed.2017.08.017>.

55. Dlamini-Simelane TT, Moyer E. 'Lost to follow up': rethinking delayed and interrupted HIV treatment among married Swazi women. Health Policy Plan. 2017;32(2):248-56. Epub 2017/02/17. doi: 10.1093/heapol/czw117. PubMed PMID: 28207052.

56. Hussen SA, Argaw MG, Tsegaye M, Andes KL, Gilliard D, Del Rio C. Gender, power and intimate relationships over the life course among Ethiopian female peer educators living with HIV. Cult Health Sex. 2019;21(4):447-61. doi: <https://dx.doi.org/10.1080/13691058.2018.1487999>.

57. Fords GM, Crowley T, van der Merwe AS. The lived experiences of rural women diagnosed with the human immunodeficiency virus in the antenatal period. Sahara j. 2017;14(1):85-92. doi: <https://dx.doi.org/10.1080/17290376.2017.1379430>.

58. Gill MM, Umutoni A, Hoffman HJ, Ndatimana D, Ndayisaba GF, Kibitenga S, et al. Understanding Antiretroviral Treatment Adherence Among HIV-Positive Women at Four Postpartum Time Intervals: Qualitative Results from the Kabeho Study in Rwanda. AIDS Patient Care STDS. 2017;31(4):153-66. doi: <https://dx.doi.org/10.1089/apc.2016.0234>.

59. Heerink F, Krumeich A, Feron F, Goga A. 'We are the advocates for the babies' - understanding interactions between patients and health care providers during the prevention of mother-to-child transmission of HIV in South Africa: a qualitative study. Glob Health Action. 2019;12(1):1630100. doi: <https://dx.doi.org/10.1080/16549716.2019.1630100>.

60. Katirayi L, Chadambuka A, Muchedzi A, Ahimbisibwe A, Musarandega R, Woelk G, et al. Echoes of old HIV paradigms: reassessing the problem of engaging men in HIV testing and treatment through women's perspectives. Reprod Health. 2017;14(1):124. doi: <https://dx.doi.org/10.1186/s12978-017-0387-1>.

61. Marais A, Kuo CC, Julies R, Stein DJ, Joska JA, Zlotnick C. "If He's Abusing You . . . the Baby Is Going to Be Affected": HIV-Positive Pregnant Women's Experiences of Intimate Partner Violence. Violence Against Women. 2019;25(7):839-61. doi: <https://dx.doi.org/10.1177/1077801218802640>.

62. McLean E, Renju J, Wamoyi J, Bukenya D, Ddaaki W, Church K, et al. 'I wanted to safeguard the baby': a qualitative study to understand the experiences of Option B+ for pregnant women and the potential implications for 'test-and-treat' in four sub-Saharan African settings. Sex Transm Infect. 2017;93(Suppl 3). Epub 2017/07/25. doi: 10.1136/sextrans-2016-052972. PubMed PMID: 28736391; PubMed Central PMCID: PMCPMC5739848.

63. Sakyi KS, Lartey MY, Dension JA, Kennedy CE, Mullany LC, Owusu PG, et al. Low Birthweight, Retention in HIV Care, and Adherence to ART Among Postpartum Women Living with HIV in Ghana. AIDS Behav. 2019;23(2):433-44. doi: <https://dx.doi.org/10.1007/s10461-018-2194-2>.

64. Spangler SA, Abuogi LL, Akama E, Bukusi EA, Helova A, Musoke P, et al. From 'half-dead' to being 'free': resistance to HIV stigma, self-disclosure and support for PMTCT/HIV care among couples living with HIV in Kenya. Cult Health Sex. 2018;20(5):489-503. doi: <https://dx.doi.org/10.1080/13691058.2017.1359338>.

65. Watt MH, Knippler ET, Knettel BA, Sikkema KJ, Ciya N, Myer L, et al. HIV Disclosure Among Pregnant Women Initiating ART in Cape Town, South Africa: Qualitative Perspectives During the Pregnancy and Postpartum Periods. AIDS Behav. 2018;22(12):3945-56. doi: <https://dx.doi.org/10.1007/s10461-018-2272-5>.

66. Conroy AA, McKenna SA, Leddy A, Johnson MO, Ngubane T, Darbes LA, et al. "If She is Drunk, I Don't Want Her to Take it": Partner Beliefs and Influence on Use of Alcohol and Antiretroviral Therapy in South African Couples. AIDS Behav. 2017;21(7):1885-91. doi: <https://dx.doi.org/10.1007/s10461-017-1697-6>.

67. Conroy A, Leddy A, Johnson M, Ngubane T, van Rooyen H, Darbes L. 'I told her this is your life': relationship dynamics, partner support and adherence to antiretroviral therapy among South African couples. Cult Health Sex. 2017;19(11):1239-53. doi: <https://dx.doi.org/10.1080/13691058.2017.1309460>.

68. Conroy AA, McKenna SA, Comfort ML, Darbes LA, Tan JY, Mkandawire J. Marital infidelity, food insecurity, and couple instability: A web of challenges for dyadic coordination around antiretroviral therapy. Soc Sci Med. 2018;214:110-7. doi: <https://dx.doi.org/10.1016/j.socscimed.2018.08.006>.

69. Conroy AA, McKenna SA, Ruark A. Couple Interdependence Impacts Alcohol Use and Adherence to Antiretroviral Therapy in Malawi. AIDS Behav. 2019;23(1):201-10. doi: <https://dx.doi.org/10.1007/s10461-018-2275-2>.

70. Conroy AA, Ruark A, McKenna SA, Tan JY, Darbes LA, Hahn JA, et al. The Unaddressed Needs of Alcohol-Using Couples on Antiretroviral Therapy in Malawi: Formative Research on Multilevel Interventions. AIDS Behav. 2019. doi: <https://dx.doi.org/10.1007/s10461-019-02653-y>.

71. Monroe-Wise A, Maingi Mutiti P, Kimani H, Moraa H, Bukusi DE, Farquhar C. Assisted partner notification services for patients receiving HIV care and treatment in an HIV clinic in Nairobi, Kenya: a qualitative assessment of barriers and opportunities for scale-up. Journal of the International AIDS Society. 2019;22 Suppl 3:e25315. doi: <https://dx.doi.org/10.1002/jia2.25315>.

72. Ware NC, Pisarski EE, Nakku-Joloba E, Wyatt MA, Muwonge TR, Turyameeba B, et al. Integrated delivery of antiretroviral treatment and pre-exposure prophylaxis to HIV-1 serodiscordant couples in East Africa: a qualitative evaluation study in Uganda. Journal of the International AIDS Society. 2018;21(5):e25113. doi: <https://dx.doi.org/10.1002/jia2.25113>.

73. Graham SM, Micheni M, Secor A, van der Elst EM, Kombo B, Operario D, et al. HIV care engagement and ART adherence among Kenyan gay, bisexual, and other men who have sex with men: a multi-level model informed by qualitative research. AIDS Care. 2018;30(sup5):S97-S105. Epub 2019/01/23. doi: 10.1080/09540121.2018.1515471. PubMed PMID: 30668136; PubMed Central PMCID: PMCPMC6430645.

74. Matovu JKB, Musinguzi G, Kiguli J, Nuwaha F, Mujisha G, Musinguzi J, et al. Health providers' experiences, perceptions and readiness to provide HIV services to men who have sex with men and female sex workers in Uganda - a qualitative study. BMC Infect Dis. 2019;19(1):214. doi: <https://dx.doi.org/10.1186/s12879-019-3713-0>.

75. Micheni M, Kombo BK, Secor A, Simoni JM, Operario D, van der Elst EM, et al. Health Provider Views on Improving Antiretroviral Therapy Adherence Among Men Who Have Sex with Men in Coastal Kenya. AIDS Patient Care STDS. 2017;31(3):113-21. doi: <https://dx.doi.org/10.1089/apc.2016.0213>.

76. Cooke A, Saleem H, Mushi D, Mbwambo J, Hassan S, Lambdin BH. Convenience without disclosure: a formative research study of a proposed integrated methadone and antiretroviral therapy service delivery model in Dar es Salaam, Tanzania. Addict Sci Clin Pract. 2017;12(1):23. doi: <https://dx.doi.org/10.1186/s13722-017-0089-6>.

77. Magidson JF, Joska JA, Regenauer KS, Satinsky E, Andersen LS, Seitz-Brown CJ, et al. "Someone who is in this thing that I am suffering from": The role of peers and other facilitators for task sharing substance use treatment in South African HIV care. Int J Drug Policy. 2019;70:61-9. doi: <https://dx.doi.org/10.1016/j.drugpo.2018.11.004>.

78. Kiplagat J, Mwangi A, Chasela C, Huschke S. Challenges with seeking HIV care services: perspectives of older adults infected with HIV in western Kenya. BMC Public Health. 2019;19(1):929. doi: <https://dx.doi.org/10.1186/s12889-019-7283-2>.

79. Schatz E, Seeley J, Negin J, Weiss HA, Tumwekwase G, Kabunga E, et al. "For us here, we remind ourselves": strategies and barriers to ART access and adherence among older Ugandans. BMC Public Health. 2019;19(1):131. doi: <https://dx.doi.org/10.1186/s12889-019-6463-4>.

80. Schenk KD, Tun W, Sheehy M, Okal J, Kuffour E, Moono G, et al. "Even the fowl has feelings": access to HIV information and services among persons with disabilities in Ghana, Uganda, and Zambia. Disabil Rehabil. 2018:1-14. doi: <https://dx.doi.org/10.1080/09638288.2018.1498138>.

81. Steenberg B. HIV-positive Mozambican migrants in South Africa: loneliness, secrecy and disclosure. Cult Health Sex. 2019:1-16. doi: <https://dx.doi.org/10.1080/13691058.2019.1571230>.
