# Supplementary material for: Testing for saturation in qualitative evidence syntheses: An update of HIV adherence in Africa
Source: PLoS One. 2021 Oct 19;16(10):e0258352. doi: 10.1371/journal.pone.0258352 (PMC8525762; doi:10.1371/journal.pone.0258352)
Supplement: S2 Table — (DOCX) [file pone.0258352.s002.docx]

# S2 Table: References to studies confirming, extending and enriching the findings of the original review

| **Studies confirming findings:**  **n [References to studies]** | **Studies extending themes:**  **n [References to studies]** | **Studies enriching our understanding:**  **n [References to studies]** |
| --- | --- | --- |
| **Theme 1: Poverty, competing priorities and an unpredictable microworld** | | |
| 27  [1-22] | - | 12  [1, 6, 10, 14, 16-18, 23-27] |
| **Theme 2: Social identity and gender norms can have a profound impact on care-seeking behaviour** | | |
| 33  [2, 3, 7, 9, 10, 13, 17, 18, 21, 28-52] | 9  [2, 3, 16, 31, 32, 38, 49, 50, 53] | 24  [2, 3, 7, 10, 12, 13, 21, 23, 24, 28-33, 35-37, 45, 46, 48, 49, 51, 53] |
| **Theme 3: Alienation makes it hard to take ART** | | |
| 24  [8, 9, 16-18, 20-22, 28, 29, 31, 34, 36, 39, 43, 44, 47-49, 54-59] |  | 10  [23, 29, 31, 43, 48, 49, 56-58, 60] |
| **Theme 4: People with HIV receive conflicting information, messages and views** | | |
| 20  [8, 9, 14, 19-21, 26, 28, 43-46, 48, 55, 60-65] | 5  [23, 24, 43, 48, 58] | 13  [26, 28, 39, 43, 58, 60-62, 64-68] |
| **Theme 5: “Bad patients” are an unhelpful construct of an authoritarian health** | | |
| 21  [8, 15, 16, 19, 20, 22, 28, 30, 39, 40, 42, 48, 61, 63, 66, 69-74] | 1  [7] | 12  [7, 9, 15, 30, 42, 61-63, 70, 72-74] |
| **Theme 6: Poor clinic services for patients and inadequate support for health workers** | | |
| 13  [6, 8, 16, 21, 22, 25, 34, 48, 61, 63, 72, 73, 75] | 3  [12, 69, 72] | 8  [8, 9, 12, 21, 42, 61, 63, 73] |
| **Theme 7: The new normal requires daily drugs** | | |
| 27  [1, 8, 14, 15, 19-21, 26, 28, 31-33, 45, 47, 54-58, 60, 62, 66, 71, 72, 75-77] | 7  [1, 31, 46, 47, 56, 57, 76] | 17  [1, 9, 15, 17-19, 30, 32, 39, 46, 47, 49, 55, 56, 60, 71, 76] |
| **Theme 8: Self-efficacy, social responsibility and support helps** | | |
| 42  [1, 2, 8-12, 14, 17, 18, 21, 22, 25, 27, 29-31, 39, 41, 42, 44-47, 50, 52, 54, 55, 57-61, 65, 70-76, 78, 79] | 9  [12, 22, 29, 37, 46, 49, 54, 55, 78] | 26  [2, 3, 9, 11, 12, 14, 16, 22, 25, 27, 32, 33, 36, 38, 40, 42, 48, 52, 57, 60, 65, 66, 70, 71, 77, 80] |
| **Theme 9: The tipping point** | | |
| 6  [1, 12, 15, 50, 70, 71] |  | 9  [1, 2, 14, 15, 22, 56, 70, 71, 78] |

1. Burns R, Borges J, Blasco P, Vandenbulcke A, Mukui I, Magalasi D, et al. 'I saw it as a second chance': A qualitative exploration of experiences of treatment failure and regimen change among people living with HIV on second- and third-line antiretroviral therapy in Kenya, Malawi and Mozambique. Glob Public Health. 2019;14(8):1112-24. doi: <https://dx.doi.org/10.1080/17441692.2018.1561921>.

2. Conroy A, Leddy A, Johnson M, Ngubane T, van Rooyen H, Darbes L. 'I told her this is your life': relationship dynamics, partner support and adherence to antiretroviral therapy among South African couples. Cult Health Sex. 2017;19(11):1239-53. doi: <https://dx.doi.org/10.1080/13691058.2017.1309460>.

3. Conroy AA, McKenna SA, Comfort ML, Darbes LA, Tan JY, Mkandawire J. Marital infidelity, food insecurity, and couple instability: A web of challenges for dyadic coordination around antiretroviral therapy. Soc Sci Med. 2018;214:110-7. doi: <https://dx.doi.org/10.1016/j.socscimed.2018.08.006>.

4. Czaicki NL. Understanding and informing interventions to improve antiretroviral adherence: Three papers on antiretroviral adherence in Sub-Saharan Africa. 2019;80. PubMed PMID: 2018-52508-008.

5. Czaicki NL, Mnyippembe A, Blodgett M, Njau P, McCoy SI. It helps me live, sends my children to school, and feeds me: a qualitative study of how food and cash incentives may improve adherence to treatment and care among adults living with HIV in Tanzania. AIDS Care. 2017;29(7):876-84. doi: <https://dx.doi.org/10.1080/09540121.2017.1287340>.

6. Kiplagat J, Mwangi A, Chasela C, Huschke S. Challenges with seeking HIV care services: perspectives of older adults infected with HIV in western Kenya. BMC Public Health. 2019;19(1):929. doi: <https://dx.doi.org/10.1186/s12889-019-7283-2>.

7. Knight J, Wachira J, Kafu C, Braitstein P, Wilson IB, Harrison A, et al. The Role of Gender in Patient-Provider Relationships: A Qualitative Analysis of HIV Care Providers in Western Kenya with Implications for Retention in Care. AIDS Behav. 2019;23(2):395-405. doi: <https://dx.doi.org/10.1007/s10461-018-2265-4>.

8. Mesic A, Halim N, MacLeod W, Haker C, Mwansa M, Biemba G. Facilitators and Barriers to Adherence to Antiretroviral Therapy and Retention in Care Among Adolescents Living with HIV/AIDS in Zambia: A Mixed Methods Study. AIDS Behav. 2019;23(9):2618-28. doi: <https://dx.doi.org/10.1007/s10461-019-02533-5>.

9. Micheni M, Kombo BK, Secor A, Simoni JM, Operario D, van der Elst EM, et al. Health Provider Views on Improving Antiretroviral Therapy Adherence Among Men Who Have Sex with Men in Coastal Kenya. AIDS Patient Care STDS. 2017;31(3):113-21. doi: <https://dx.doi.org/10.1089/apc.2016.0213>.

10. Murray SM, Familiar I, Nakasujja N, Winch PJ, Gallo JJ, Opoka R, et al. Caregiver mental health and HIV-infected child wellness: perspectives from Ugandan caregivers. AIDS Care. 2017;29(6):793-9. doi: <https://dx.doi.org/10.1080/09540121.2016.1263722>.

11. Nalugya R, Russell S, Zalwango F, Seeley J. The role of children in their HIV-positive parents' management of antiretroviral therapy in Uganda. Afr J AIDS Res. 2018;17(1):37-46. doi: <https://dx.doi.org/10.2989/16085906.2017.1394332>.

12. Nanfuka EK, Kyaddondo D, Ssali SN, Asingwire N. Social capital and resilience among people living on antiretroviral therapy in resource-poor Uganda. PLoS One. 2018;13(6):e0197979. Epub 2018/06/12. doi: 10.1371/journal.pone.0197979. PubMed PMID: 29889849; PubMed Central PMCID: PMCPMC5995438.

13. Naugle DA, Tibbels NJ, Hendrickson ZM, Dosso A, Van Lith L, Mallalieu EC, et al. Bringing fear into focus: The intersections of HIV and masculine gender norms in Cote d'Ivoire. PLoS One. 2019;14(10):e0223414. doi: <https://dx.doi.org/10.1371/journal.pone.0223414>.

14. Patterson AS. Engaging therapeutic citizenship and clientship: Untangling the reasons for therapeutic pacifism among people living with HIV in urban Zambia. Glob Public Health. 2016;11(9):1121-34. doi: 10.1080/17441692.2015.1070053. PubMed PMID: 117745406. Language: English. Entry Date: 20160909. Revision Date: 20190209. Publication Type: Article.

15. Renju J, Moshabela M, McLean E, Ddaaki W, Skovdal M, Odongo F, et al. 'Side effects' are 'central effects' that challenge retention in HIV treatment programmes in six sub-Saharan African countries: a multicountry qualitative study. Sex Transm Infect. 2017;93(Suppl 3). doi: <https://dx.doi.org/10.1136/sextrans-2016-052971>.

16. Schatz E, Seeley J, Negin J, Weiss HA, Tumwekwase G, Kabunga E, et al. "For us here, we remind ourselves": strategies and barriers to ART access and adherence among older Ugandans. BMC Public Health. 2019;19(1):131. doi: <https://dx.doi.org/10.1186/s12889-019-6463-4>.

17. Sileo KM, Kizito W, Wanyenze RK, Chemusto H, Musoke W, Mukasa B, et al. A qualitative study on alcohol consumption and HIV treatment adherence among men living with HIV in Ugandan fishing communities. AIDS Care. 2019;31(1):35-40. doi: <https://dx.doi.org/10.1080/09540121.2018.1524564>.

18. Sileo KM, Reed E, Kizito W, Wagman JA, Stockman JK, Wanyenze RK, et al. Masculinity and engagement in HIV care among male fisherfolk on HIV treatment in Uganda. Cult Health Sex. 2019;21(7):774-88. doi: <https://dx.doi.org/10.1080/13691058.2018.1516299>.

19. Skovdal M, Wringe A, Seeley J, Renju J, Paparini S, Wamoyi J, et al. Using theories of practice to understand HIV-positive persons varied engagement with HIV services: a qualitative study in six Sub-Saharan African countries. Sex Transm Infect. 2017;93(Suppl 3). Epub 2017/07/25. doi: 10.1136/sextrans-2016-052977. PubMed PMID: 28736396; PubMed Central PMCID: PMCPMC5739842.

20. Stern E, Colvin C, Gxabagxaba N, Schutz C, Burton R, Meintjes G. Conceptions of agency and constraint for HIV-positive patients and healthcare workers to support long-term engagement with antiretroviral therapy care in Khayelitsha, South Africa. Afr J AIDS Res. 2017;16(1):19-29. doi: <https://dx.doi.org/10.2989/16085906.2017.1285795>.

21. Tibbels NJ, Hendrickson ZM, Naugle DA, Dosso A, Van Lith L, Mallalieu EC, et al. Men's perceptions of HIV care engagement at the facility- and provider-levels: Experiences in Cote d'Ivoire. PLoS One. 2019;14(3):e0211385. doi: <https://dx.doi.org/10.1371/journal.pone.0211385>.

22. Topp SM, Mwamba C, Sharma A, Mukamba N, Beres LK, Geng E, et al. Rethinking retention: Mapping interactions between multiple factors that influence long-term engagement in HIV care. PLoS One. 2018;13(3):e0193641. doi: <https://dx.doi.org/10.1371/journal.pone.0193641>.

23. Kimera E, Vindevogel S, Rubaihayo J, Reynaert D, De Maeyer J, Engelen A-M, et al. Youth living with HIV/AIDS in secondary schools: perspectives of peer educators and patron teachers in Western Uganda on stressors and supports. Sahara j. 2019;16(1):51-61. doi: <https://dx.doi.org/10.1080/17290376.2019.1626760>.

24. Mackworth-Young CR, Bond V, Wringe A, Konayuma K, Clay S, Chiiya C, et al. "My mother told me that I should not": a qualitative study exploring the restrictions placed on adolescent girls living with HIV in Zambia. Journal of the International AIDS Society. 2017;20(4). doi: <https://dx.doi.org/10.1002/jia2.25035>.

25. Sakyi KS, Lartey MY, Dension JA, Kennedy CE, Mullany LC, Owusu PG, et al. Low Birthweight, Retention in HIV Care, and Adherence to ART Among Postpartum Women Living with HIV in Ghana. AIDS Behav. 2019;23(2):433-44. doi: <https://dx.doi.org/10.1007/s10461-018-2194-2>.

26. Tocco JU. The Islamification of antiretroviral therapy: Reconciling HIV treatment and religion in northern Nigeria. Soc Sci Med. 2017;190:75-82. doi: <https://dx.doi.org/10.1016/j.socscimed.2017.08.017>.

27. Zuma T, Wight D, Rochat T, Moshabela M. Traditional health practitioners' management of HIV/AIDS in rural South Africa in the era of widespread antiretroviral therapy. Glob Health Action. 2017;10(1):1352210. doi: 10.1080/16549716.2017.1352210. PubMed PMID: 28771116.

28. Adams AK, Zamberia AM. "I will take ARVs once my body deteriorates": an analysis of Swazi men's perceptions and acceptability of Test and Start. Afr J AIDS Res. 2017;16(4):295-303. doi: <https://dx.doi.org/10.2989/16085906.2017.1362015>.

29. Ashaba S, Cooper-Vince CE, Vorechovska D, Rukundo GZ, Maling S, Akena D, et al. Community beliefs, HIV stigma, and depression among adolescents living with HIV in rural Uganda. Afr J AIDS Res. 2019;18(3):169-80. doi: <https://dx.doi.org/10.2989/16085906.2019.1637912>.

30. Bernays S, Paparini S, Seeley J, Rhodes T. "Not Taking it Will Just be Like a Sin": Young People Living with HIV and the Stigmatization of Less-Than-Perfect Adherence to Antiretroviral Therapy. Med Anthropol. 2017;36(5):485-99. doi: <https://dx.doi.org/10.1080/01459740.2017.1306856>.

31. Bonnington O, Wamoyi J, Ddaaki W, Bukenya D, Ondenge K, Skovdal M, et al. Changing forms of HIV-related stigma along the HIV care and treatment continuum in sub-Saharan Africa: a temporal analysis. Sex Transm Infect. 2017;93(Suppl 3). doi: <https://dx.doi.org/10.1136/sextrans-2016-052975>.

32. Conroy AA, McKenna SA, Ruark A. Couple Interdependence Impacts Alcohol Use and Adherence to Antiretroviral Therapy in Malawi. AIDS Behav. 2019;23(1):201-10. doi: <https://dx.doi.org/10.1007/s10461-018-2275-2>.

33. Conroy AA, Ruark A, McKenna SA, Tan JY, Darbes LA, Hahn JA, et al. The Unaddressed Needs of Alcohol-Using Couples on Antiretroviral Therapy in Malawi: Formative Research on Multilevel Interventions. AIDS Behav. 2019. doi: <https://dx.doi.org/10.1007/s10461-019-02653-y>.

34. Cooke A, Saleem H, Mushi D, Mbwambo J, Hassan S, Lambdin BH. Convenience without disclosure: a formative research study of a proposed integrated methadone and antiretroviral therapy service delivery model in Dar es Salaam, Tanzania. Addict Sci Clin Pract. 2017;12(1):23. doi: <https://dx.doi.org/10.1186/s13722-017-0089-6>.

35. Dlamini-Simelane TT, Moyer E. 'Lost to follow up': rethinking delayed and interrupted HIV treatment among married Swazi women. Health Policy Plan. 2017;32(2):248-56. Epub 2017/02/17. doi: 10.1093/heapol/czw117. PubMed PMID: 28207052.

36. Enane LA, Apondi E, Toromo J, Bosma C, Ngeresa A, Nyandiko W, et al. "A problem shared is half solved" - a qualitative assessment of barriers and facilitators to adolescent retention in HIV care in western Kenya. AIDS Care. 2019:1-9. doi: <https://dx.doi.org/10.1080/09540121.2019.1668530>.

37. Graham SM, Micheni M, Secor A, van der Elst EM, Kombo B, Operario D, et al. HIV care engagement and ART adherence among Kenyan gay, bisexual, and other men who have sex with men: a multi-level model informed by qualitative research. AIDS Care. 2018;30(sup5):S97-S105. Epub 2019/01/23. doi: 10.1080/09540121.2018.1515471. PubMed PMID: 30668136; PubMed Central PMCID: PMCPMC6430645.

38. Hussen SA, Argaw MG, Tsegaye M, Andes KL, Gilliard D, Del Rio C. Gender, power and intimate relationships over the life course among Ethiopian female peer educators living with HIV. Cult Health Sex. 2019;21(4):447-61. doi: <https://dx.doi.org/10.1080/13691058.2018.1487999>.

39. Madhombiro M, Marimbe-Dube B, Dube M, Kaiyo-Utete M, Paradzai A, Chibanda D, et al. Perceptions of alcohol use in the context of HIV treatment: a qualitative study. HIV AIDS (Auckl). 2018;10:47-55. doi: <https://dx.doi.org/10.2147/HIV.S150095>.

40. Magidson JF, Joska JA, Regenauer KS, Satinsky E, Andersen LS, Seitz-Brown CJ, et al. "Someone who is in this thing that I am suffering from": The role of peers and other facilitators for task sharing substance use treatment in South African HIV care. Int J Drug Policy. 2019;70:61-9. doi: <https://dx.doi.org/10.1016/j.drugpo.2018.11.004>.

41. Marais A, Kuo CC, Julies R, Stein DJ, Joska JA, Zlotnick C. "If He's Abusing You . . . the Baby Is Going to Be Affected": HIV-Positive Pregnant Women's Experiences of Intimate Partner Violence. Violence Against Women. 2019;25(7):839-61. doi: <https://dx.doi.org/10.1177/1077801218802640>.

42. Matovu JKB, Musinguzi G, Kiguli J, Nuwaha F, Mujisha G, Musinguzi J, et al. Health providers' experiences, perceptions and readiness to provide HIV services to men who have sex with men and female sex workers in Uganda - a qualitative study. BMC Infect Dis. 2019;19(1):214. doi: <https://dx.doi.org/10.1186/s12879-019-3713-0>.

43. McHenry MS, Nyandiko WM, Scanlon ML, Fischer LJ, McAteer CI, Aluoch J, et al. HIV Stigma: Perspectives from Kenyan Child Caregivers and Adolescents Living with HIV. J Int Assoc Provid AIDS Care. 2017;16(3):215-25. doi: <https://dx.doi.org/10.1177/2325957416668995>.

44. Monroe-Wise A, Maingi Mutiti P, Kimani H, Moraa H, Bukusi DE, Farquhar C. Assisted partner notification services for patients receiving HIV care and treatment in an HIV clinic in Nairobi, Kenya: a qualitative assessment of barriers and opportunities for scale-up. Journal of the International AIDS Society. 2019;22 Suppl 3:e25315. doi: <https://dx.doi.org/10.1002/jia2.25315>.

45. Mutumba M, Musiime V, Mugerwa H, Nakyambadde H, Gautam A, Matama C, et al. Perceptions of HIV Self-Management Roles and Challenges in Adolescents, Caregivers, and Health Care Providers. JANAC: Journal of the Association of Nurses in AIDS Care. 2019;30(4):415-27. doi: 10.1097/JNC.0000000000000011. PubMed PMID: 139218567. Language: English. Entry Date: 20191031. Revision Date: 20191031. Publication Type: Article. Journal Subset: Core Nursing.

46. Rosenbaum L. Exploring the social ecological actors that contribute to the resilience of adolescents living with HIV in South Africa: A photovoice study. 2018;78. PubMed PMID: 2017-43830-104.

47. Russell S. Men's Refashioning of Masculine Identities in Uganda and Their Self-Management of HIV Treatment. Qual Health Res. 2019;29(8):1199-212. doi: <https://dx.doi.org/10.1177/1049732318823717>.

48. Schenk KD, Tun W, Sheehy M, Okal J, Kuffour E, Moono G, et al. "Even the fowl has feelings": access to HIV information and services among persons with disabilities in Ghana, Uganda, and Zambia. Disabil Rehabil. 2018:1-14. doi: <https://dx.doi.org/10.1080/09638288.2018.1498138>.

49. Sikstrom L. 'He is almost like other children': An ethnography of Malawi's national pediatric HIV treatment programme. 2017;77. PubMed PMID: 2016-47709-036.

50. Wamoyi J, Renju J, Moshabela M, McLean E, Nyato D, Mbata D, et al. Understanding the relationship between couple dynamics and engagement with HIV care services: insights from a qualitative study in Eastern and Southern Africa. Sex Transm Infect. 2017;93(Suppl 3). doi: <https://dx.doi.org/10.1136/sextrans-2016-052976>.

51. Woollett N, Black V, Cluver L, Brahmbhatt H. Reticence in disclosure of HIV infection and reasons for bereavement: impact on perinatally infected adolescents' mental health and understanding of HIV treatment and prevention in Johannesburg, South Africa. Afr J AIDS Res. 2017;16(2):175-84. doi: <https://dx.doi.org/10.2989/16085906.2017.1337646>.

52. Zanoni BC, Sibaya T, Cairns C, Haberer JE. Barriers to Retention in Care are Overcome by Adolescent-Friendly Services for Adolescents Living with HIV in South Africa: A Qualitative Analysis. AIDS Behav. 2019;23(4):957-65. doi: <https://dx.doi.org/10.1007/s10461-018-2352-6>.

53. Katirayi L, Chadambuka A, Muchedzi A, Ahimbisibwe A, Musarandega R, Woelk G, et al. Echoes of old HIV paradigms: reassessing the problem of engaging men in HIV testing and treatment through women's perspectives. Reprod Health. 2017;14(1):124. doi: <https://dx.doi.org/10.1186/s12978-017-0387-1>.

54. Earnshaw VA, Bogart LM, Courtney I, Zanoni H, Bangsberg DR, Orrell C, et al. Exploring Treatment Needs and Expectations for People Living with HIV in South Africa: A Qualitative Study. AIDS Behav. 2018;22(8):2543-52. doi: 10.1007/s10461-018-2101-x. PubMed PMID: 29619585.

55. Gill MM, Umutoni A, Hoffman HJ, Ndatimana D, Ndayisaba GF, Kibitenga S, et al. Understanding Antiretroviral Treatment Adherence Among HIV-Positive Women at Four Postpartum Time Intervals: Qualitative Results from the Kabeho Study in Rwanda. AIDS Patient Care STDS. 2017;31(4):153-66. doi: <https://dx.doi.org/10.1089/apc.2016.0234>.

56. Horter S, Bernays S, Thabede Z, Dlamini V, Kerschberger B, Pasipamire M, et al. "I don't want them to know": how stigma creates dilemmas for engagement with Treat-all HIV care for people living with HIV in Eswatini. Afr J AIDS Res. 2019;18(1):27-37. doi: <https://dx.doi.org/10.2989/16085906.2018.1552163>.

57. Spangler SA, Abuogi LL, Akama E, Bukusi EA, Helova A, Musoke P, et al. From 'half-dead' to being 'free': resistance to HIV stigma, self-disclosure and support for PMTCT/HIV care among couples living with HIV in Kenya. Cult Health Sex. 2018;20(5):489-503. doi: <https://dx.doi.org/10.1080/13691058.2017.1359338>.

58. Steenberg B. HIV-positive Mozambican migrants in South Africa: loneliness, secrecy and disclosure. Cult Health Sex. 2019:1-16. doi: <https://dx.doi.org/10.1080/13691058.2019.1571230>.

59. Watt MH, Knippler ET, Knettel BA, Sikkema KJ, Ciya N, Myer L, et al. HIV Disclosure Among Pregnant Women Initiating ART in Cape Town, South Africa: Qualitative Perspectives During the Pregnancy and Postpartum Periods. AIDS Behav. 2018;22(12):3945-56. doi: <https://dx.doi.org/10.1007/s10461-018-2272-5>.

60. Burman C, Aphane M. Improved adherence to anti-retroviral therapy among traditionalists: reflections from rural South Africa. Afr Health Sci. 2019;19(1):1422-32. doi: <https://dx.doi.org/10.4314/ahs.v19i1.15>.

61. Heerink F, Krumeich A, Feron F, Goga A. 'We are the advocates for the babies' - understanding interactions between patients and health care providers during the prevention of mother-to-child transmission of HIV in South Africa: a qualitative study. Glob Health Action. 2019;12(1):1630100. doi: <https://dx.doi.org/10.1080/16549716.2019.1630100>.

62. Moshabela M, Bukenya D, Darong G, Wamoyi J, McLean E, Skovdal M, et al. Traditional healers, faith healers and medical practitioners: the contribution of medical pluralism to bottlenecks along the cascade of care for HIV/AIDS in Eastern and Southern Africa. Sexually Transmitted Infections. 2017;93(Suppl 3). doi: 10.1136/sextrans-2016-052974.

63. Mwamba C, Sharma A, Mukamba N, Beres L, Geng E, Holmes CB, et al. 'They care rudely!': resourcing and relational health system factors that influence retention in care for people living with HIV in Zambia. BMJ global health. 2018;3(5):e001007. doi: <https://dx.doi.org/10.1136/bmjgh-2018-001007>.

64. Rubincam C. "It's natural to look for a source": A qualitative examination of alternative beliefs about HIV and AIDS in Cape Town, South Africa. Public understanding of science (Bristol, England). 2017;26(3):369-84. doi: <https://dx.doi.org/10.1177/0963662515611823>.

65. Zuma T, Wight D, Rochat T, Moshabela M. Navigating Multiple Sources of Healing in the Context of HIV/AIDS and Wide Availability of Antiretroviral Treatment: A Qualitative Study of Community Participants' Perceptions and Experiences in Rural South Africa. Frontiers in public health. 2018;6:73. doi: <https://dx.doi.org/10.3389/fpubh.2018.00073>.

66. Conroy AA, McKenna SA, Leddy A, Johnson MO, Ngubane T, Darbes LA, et al. "If She is Drunk, I Don't Want Her to Take it": Partner Beliefs and Influence on Use of Alcohol and Antiretroviral Therapy in South African Couples. AIDS Behav. 2017;21(7):1885-91. doi: <https://dx.doi.org/10.1007/s10461-017-1697-6>.

67. Ntela S-DM, Goutte N, Morvillers J-M, Crozet C, Ahouah M, Omanyondo-Ohambe M-C, et al. Observance to antiretroviral treatment in the rural region of the Democratic Republic of Congo: a cognitive dissonance. Pan Afr Med J. 2018;31:159. doi: <https://dx.doi.org/10.11604/pamj.2018.31.159.15132>.

68. Weintraub A, Mantell JE, Holt K, Street RA, Wilkey C, Dawad S, et al. 'These people who dig roots in the forests cannot treat HIV': Women and men in Durban, South Africa, reflect on traditional medicine and antiretroviral drugs. Glob Public Health. 2018;13(1):115-27. doi: 10.1080/17441692.2017.1359326. PubMed PMID: 28793809.

69. Harris B, Eyles J, Goudge J. Ways of doing: Restorative practices, governmentality, and provider conduct in post-apartheid health care. Med Anthropol. 2016;35(6):572-87. doi: 10.1080/01459740.2016.1173691. PubMed PMID: 2016-54064-011.

70. Hurley EA, Harvey SA, Winch PJ, Keita M, Roter DL, Doumbia S, et al. The Role of Patient-Provider Communication in Engagement and Re-engagement in HIV Treatment in Bamako, Mali: A Qualitative Study. J Health Commun. 2018;23(2):129-43. doi: <https://dx.doi.org/10.1080/10810730.2017.1417513>.

71. McLean E, Renju J, Wamoyi J, Bukenya D, Ddaaki W, Church K, et al. 'I wanted to safeguard the baby': a qualitative study to understand the experiences of Option B+ for pregnant women and the potential implications for 'test-and-treat' in four sub-Saharan African settings. Sex Transm Infect. 2017;93(Suppl 3). Epub 2017/07/25. doi: 10.1136/sextrans-2016-052972. PubMed PMID: 28736391; PubMed Central PMCID: PMCPMC5739848.

72. Mulqueeny DM, Taylor M. Does the public antiretroviral treatment programme meet patients' needs? A study at four hospitals in eThekwini, KwaZulu-Natal, South Africa. Afr J Prim Health Care Fam Med. 2019;11(1):e1-e11. doi: <https://dx.doi.org/10.4102/phcfm.v11i1.1824>.

73. Ondenge K, Renju J, Bonnington O, Moshabela M, Wamoyi J, Nyamukapa C, et al. 'I am treated well if I adhere to my HIV medication': putting patient-provider interactions in context through insights from qualitative research in five sub-Saharan African countries. Sex Transm Infect. 2017;93(Suppl 3). Epub 2017/07/25. doi: 10.1136/sextrans-2016-052973. PubMed PMID: 28736392; PubMed Central PMCID: PMCPMC5739840.

74. Wringe A, Moshabela M, Nyamukapa C, Bukenya D, Ondenge K, Ddaaki W, et al. HIV testing experiences and their implications for patient engagement with HIV care and treatment on the eve of 'test and treat': findings from a multicountry qualitative study. Sex Transm Infect. 2017;93(Suppl 3). doi: <https://dx.doi.org/10.1136/sextrans-2016-052969>.

75. Fords GM, Crowley T, van der Merwe AS. The lived experiences of rural women diagnosed with the human immunodeficiency virus in the antenatal period. Sahara j. 2017;14(1):85-92. doi: <https://dx.doi.org/10.1080/17290376.2017.1379430>.

76. Hendrickson ZM, Naugle DA, Tibbels N, Dosso A, M Van Lith L, Mallalieu EC, et al. "You Take Medications, You Live Normally": The Role of Antiretroviral Therapy in Mitigating Men's Perceived Threats of HIV in Cote d'Ivoire. AIDS Behav. 2019;23(9):2600-9. doi: <https://dx.doi.org/10.1007/s10461-019-02614-5>.

77. Ware NC, Pisarski EE, Nakku-Joloba E, Wyatt MA, Muwonge TR, Turyameeba B, et al. Integrated delivery of antiretroviral treatment and pre-exposure prophylaxis to HIV-1 serodiscordant couples in East Africa: a qualitative evaluation study in Uganda. Journal of the International AIDS Society. 2018;21(5):e25113. doi: <https://dx.doi.org/10.1002/jia2.25113>.

78. Siril H, Fawzi MCS, Todd J, Wyatt M, Kilewo J, Ware N, et al. Hopefulness Fosters Affective and Cognitive Constructs for Actions to Cope and Enhance Quality of Life among People Living with HIV in Dar Es Salaam, Tanzania. J Int Assoc Provid AIDS Care. 2017;16(2):140-8. doi: <https://dx.doi.org/10.1177/2325957414539195>.

79. Skovdal M, Maswera R, Kadzura N, Nyamukapa C, Rhead R, Wringe A, et al. Parental obligations, care and HIV treatment: How care for others motivates self-care in Zimbabwe. J Health Psychol. 2018:1359105318788692. doi: <https://dx.doi.org/10.1177/1359105318788692>.

80. Wachira J, Genberg B, Kafu C, Braitstein P, Laws MB, Wilson IB. Experiences and expectations of patients living with HIV on their engagement with care in Western Kenya. Patient Prefer Adherence. 2018;12:1393-400. doi: <https://dx.doi.org/10.2147/PPA.S168664>.
