# Supplementary material for: Testing for saturation in qualitative evidence syntheses: An update of HIV adherence in Africa
Source: PLoS One. 2021 Oct 19;16(10):e0258352. doi: 10.1371/journal.pone.0258352 (PMC8525762; doi:10.1371/journal.pone.0258352)
Supplement: S3 Table — (DOCX) [file pone.0258352.s003.docx]

## S3 Table: List of existing and updated themes, subthemes and codes

*New codes are listed in red*

| **Theme 1: Poverty, competing priorities and an unpredictable microworld** | | | |
| --- | --- | --- | --- |
| **Sub-theme** | **Codes** | **Sub-Code 1** | **Sub-Code 2** |
| Poverty and the need to prioritize | Economic constraints | Need to earn money instead of attend clinic or support groups |  |
|  |  | Food insecurity | Adverse events potentiated by hunger leads some to stop ART |
|  |  |  | Increased appetite on ART may be a challenge for household |
|  |  | Cost of travel and services | Limited mobility of older persons |
|  | Safety concerns |  |  |
|  | The need to maintain a social life | Social and family responsibilities |  |
|  |  | Social and family network may be essential for survival |  |
|  |  | Prioritising alcohol |  |
|  | Going to school vs. going to clinic |  |  |
|  | religious vs therapeutic obligations |  |  |
|  | Comorbidities and other illnesses |  |  |
| Unpredictable life events | High mobility and unexpected travel disruptions |  |  |
|  | Unexpected change in routines |  |  |
|  | Sudden loss of support system |  |  |
|  | Political unrest and refugee status |  |  |
|  | Treatment failure |  |  |
|  | Sudden illness |  |  |
|  | Having to care for sick children | Long hospital stays |  |
|  |  | Caregiver burden |  |

| **Theme 2: Social identity and gender norms can have a profound impact on care-seeking behavior** |
| --- |

| **Sub-theme** | **Codes** | **Sub-Code 1** | **Sub-Code 2** |
| --- | --- | --- | --- |

| **Gender roles interact with HIV care** | Masculinity is at odds with HIV care | Hegemonic features of masculinity | Strong, powerful and brave, risk-takers |
| --- | --- | --- | --- |
|  |  |  | Respected in society |
|  |  |  | Provide economically for their families |
|  |  |  | Father children and have a family |
|  |  | Interaction between masculinity and HIV | HIV may erode masculine identity |
|  |  |  | Loss of sexual freedom |
|  |  |  | Mental health |
|  |  |  | Attending HIV clinics is at odds with masculine identity |
|  |  | Patriarchy | Conferment of social power and status to males and expectation of female submission |
|  | Women often have limited choices | Options to choose from are not always feasible |  |
|  |  |  |  |
|  |  |  |  |
|  |  | Economic dependency results in limited choices | Vulnerability due to dependence on husbands/partners |
|  |  |  | Poor women are more dependent on family and social network for economic, logistic and emotional help |
|  |  | Unsupportive partners, IPV and sexual abuse impacts mental health, health decisions and access to care | Unsupportive or obstructive partners |
|  |  |  | Fear of IPV |
|  |  |  | IPV and sexual abuse |
|  |  |  | Depression and hopelessness |
|  |  | Complying with HIV guidelines is challenging while maintaining gender roles in households and families | Women struggle to reduce family size |
|  |  |  | Women may choose rather to maintain family/social life if taking ART could threaten this |
|  |  |  | Women want to protect families and encourage healthy lifestyle |
|  |  | Women have defined social roles | Women want to have children and society expects it |
|  |  |  | Women are expected to care for others and may not put themselves or their health first |
|  |  |  | Need permission from others before making health decisions |
|  | Relationship dynamics influence care seeking behaviours | Idea of a shared future | Relationships take priority over ART |
|  |  |  | Desire for long-term relationships and marriage |
|  |  |  | Shared experience of ART improves relationship dynamics |
|  |  |  | Children motivates to stay together |
|  |  | Power dynamics in relationships | Women lose power if they are HIV positive |
|  |  |  | Women have more power if men brought HIV into relationship |
|  |  |  | Women gain power and independence when HIV positive partner dies |
|  |  |  | Shared power between partners (sero-concordant couples) |
|  |  | Disclosure and blame | ART causes conflicts |
|  |  |  | Verbal abuse when missing doses |
| **It is confusing and isolating being a child or adolescent with HIV:** | Influence of developmental stage | Strong desire to be like other “normal” children/adolescents |  |
|  |  | Anger, rebellion and confusion |  |
|  |  | Adolescents want to have a future |  |
|  |  | Adolescents struggle to negotiate sexual relationships with HIV |  |
|  | Highly dependent on support from others | Need stable home and school environments to thrive | Role models to self-manage |
|  |  | There is often a lot of chaos and instability in their support systems |  |
|  | Children and adolescent are at risk of feeling isolated and depressed | HIV positive children/adolescents experience a lot of losses | Loss of parents |
|  |  |  | Loss of social environment, |
|  |  |  | Incomplete grief |
|  |  | Trauma and abuse | Violence and sexual assault |
|  |  |  | Abuse and neglect by parents |
|  |  | Depression and suicidiality due to HIV diagnosis | self-hatred, anger, |
|  |  | Disempowerment |  |
|  | Adults often do not know how to deal with children and adolescents | Disclosure and lack of illness knowledge | Caretakers find it hard to disclose their own HIV status or the status of adolescents |
|  |  |  | Social norms may influence communications |
|  |  |  | Method of disclosure |
|  |  | Combination of poor communication and developmental stage can result in confusion and fear about HIV and impair ART adherence | Culture of silence |
|  |  |  | Health workers may not communicate directly or discuss concerns |
|  |  | Transfer to general or adult clinics is difficult |  |
|  |  | HCWs may hold adolescents responsible for infringements |  |
|  | Side effects and poor palatability of formulations |  |  |
|  | Fear of rejection and stigma contribute to poor adherence and engagement in care | Stigma, the desire to maintain status a secret and lack of privacy impairs adherence and engagement in care | Feeling left out |
| **HIV-positive key populations must face stigma related both to HIV and lifestyle choice** | Lack of conforming to social norms and leads to moral judgement from others |  |  |
|  | Discrimination related to lifestyle is often worse than HIV stigma |  |  |
|  | Psychological distress related to MSM lifestyle can be additional burden |  |  |
|  | Lifestyle often associated with criminality which encourages discrimination |  |  |
|  | Loss of support |  |  |
|  | For PWID sometimes addiction trumps any other needs |  |  |
|  | Key populations avoid health facilities and need a lot of help to engage in care | Discrimination keeps key populations out of HIV care |  |
|  |  | Key populations need a lot of support and health care workers that don’t discriminate to engage in HIV care |  |
| **HIV positive people with disabilities experience multiple stigmas and discrimination** | Discrimination due to disability | People with disabilities are assumed to not be sexual and thus HIV free |  |
|  | Difficulty accessing care and need consistent support |  |  |
|  | Accepting HIV and a recent disability is difficult |  |  |
|  | PWD may choose to avoid attending general clinics due to discrimination |  |  |
|  | Vulnerability due to sexual, physical, verbal and emotional abuse |  |  |
| **Older people with HIV feel insecure, outnumbered and are 'considered already dead'** | Treated differently in the clinic |  |  |
|  | Stigma is a barrier |  |  |

| **Theme 3: Alienation makes it hard to take ART** | | |
| --- | --- | --- |
| **Sub-theme** | **Codes** | **Sub-Code 1** |
| **HIV Stigma and discrimination undermines sense of belonging which impairs adherence and engagement in care** | HIV stigma is related to promiscuity, infectiousness, sickness and death |  |
|  | Stigma comes in various forms | Anticipated stigma – feeling of shame, humiliation, suicidal ideations |
|  |  | Perceived Stigma - Adolescents fear social isolation, losing friends, diminished social interactions and loss of respect, loss of material support like housing, food, employment |
|  |  | Courtesy stigma - people automatically assume caregivers of HIV+ adolescents are HIV+ too |
|  | HIV Stigma results in judgement and discrimination by others | Discrimination can may come from; peers, partners, employers, relatives, educators, community or household members |
|  |  | Discrimination can lead to loss of support |
|  |  | Discrimination can affect economic opportunities |
|  |  | Community sees adolescents with HIV as a threat |
|  |  | Older people mocked and considered already dead |
|  |  | Complexity of stigma in people with disabilities - compounded stigma of disability and HIV. |
|  |  | Migrants feel extra vulnerable due to job security, language barriers, cultural differences and medical plurism |
|  | Choosing biomedical above traditional approach alienates people |  |
|  | A wide range of complicated emotional responses undermines sense of belonging | shame, fear, confusion, guilt, anger and grief |
|  | HIV stigma can lead to people feeling socially dead | HIV stigma can make people feel ashamed and blame themselves |
|  |  | HIV positive people may feel isolated and depressed |
|  |  | HIV is a biosocial disease - changes in clinical (and knowledge) and social perceptions of the disease. 'fear of physical death' is replaced by 'fear of social death'. |
|  | Loss of support systems, hopelessness and depression may impair ART adherence and engagement in care |  |
| **HIV-positive people fear inadvertent disclosure and may disengage or skip doses if confidentiality is at risk** | HIV-positive people will work hard to maintain HIV status a secret | to avoid being gossiped about, mocked, experience social judgement, loss of reputation |
|  |  | Early initiation of ART enables people to hide status |
|  |  | Fear that asymptomatic PLHIV on ART will develop side-effects which will expose their status |
|  | It is hard to take ART If household members/partner/schoolmates are not informed |  |
|  | Lack of confidentiality and privacy | If clinic staff, location, layout or method of integration compromises confidentiality patients may disengage |
|  |  | Education system not providing privacy to take ART |
| **Theme 4: People with HIV receive conflicting information, messages and views** | | |
| **Sub-theme** | **Codes** | **Sub-Code 1** |
| **Conflicting information** | Teachers | HIV is a death sentence; “if you get HIV you are finished” |
|  | Healthcare providers | Don’t have sex |
|  |  | Don’t talk about sexuality |
|  | Parents | Don’t talk about sexuality |
| **Access to information** | People living with disabilities are unable to access information |  |
|  | Alternative discourses about HIV & ART | There are many different explanations for the causes and treatment of HIV |
|  |  | There are many different sources of information about HIV which may influence patients’ decisions |
| **Cognitive dissonance and individual beliefs influence engagement in HIV care** | Side-effects undermine the message of better health on ART | Side-effects create doubt about ART, particularly for the well |
|  |  | Some fear negative ART effects and fail to link |
|  | Scientific uncertainty creates confusion and doubt | Confusion about origin of the disease |
|  |  | Scientists are powerful and should have come up with a cure – belief that it is being withheld |
|  | People choose ideology which suits them best | Attending traditional healers is often favoured over the biomedical health system |
|  |  | Some feel that traditional healers are dishonest, ineffective and only care about profits |
|  |  | Some favour biomedical approach due to cost and belief that ART is effective |
|  |  | Ideologies which offer a cure are particularly desirable and may influence engagement in HIV care |
|  |  | Choice of healthcare provider often depends on cultural and family traditions |
|  | Medical plurism | Choose from a wide range of options including biomedical, traditional, faith-based |
|  |  | Some people switch options if they get stuck |
|  |  | Some people mix treatments e.g. ART with traditional medicine |
|  |  | Religious views in conflict with biomedical rationale |
|  | Perception of illness is complex | Shaped by past and family experiences |
|  |  | Distinction between illness emerging from within the body or outside |
|  | HCWs believe that some patients have ‘wrong beliefs’ |  |
|  | Misinformation alters beliefs | ART seen as a way of colonisation |
|  |  | There is no cure for HIV |
|  |  | HIV is a death sentence |
|  | Interactive toxicity beliefs | Belief that mixing alcohol and ART is toxic |
| **HIV is a biosocial disease** | Increased knowledge about HIV over time | Knowledge about HIV changes and is context specific |
|  |  | Social perception of disease does not change as quickly |
|  | Changes in clinical (and knowledge) and social perceptions of the disease. 'fear of physical death' is replaced by 'fear of social death'. Biosocial ambiance is dynamic but changes in understanding of illness (no longer a death sentence) and changes in social perceptions take time to change and are dependent on time and place. |  |
| **Theme 5: “Bad patients” are an unhelpful construct of an authoritarian health** | | |
| **Sub-theme** | **Codes** | **Sub-Code 1** |
| The biomedical approach is authoritarian, hierarchical and paternalistic | Biomedical system does not acknowledge medical plurism |  |
|  | Power imbalance between HCWs and HIV positive people | Adolescents see HCWs as parents |
|  |  | Health care workers hold all power at clinic |
|  |  | Patients see HCWs as powerful and try to please them |
|  |  | HCWs may abuse their powerful role and disrespect patients |
|  |  | HCWs can be judgemental and discriminate if they don’t agree with lifestyle choices |
|  |  | Guidelines may be presented as rules |
|  |  | People work hard to carry favour with HCWs and facilitate care |
|  |  | Adolescents want to maintain relationship with HCWs rather than tell the truth about adherence |
|  |  | HCWs see themselves as the ‘knowledgeable healers’ and the patients as the ‘ignorant sick’ |
|  | Patients want to be part of the decision-making processes | Patients feel that their voices are not being heard |
|  |  | Patients participate in a lot of research but do not see any impact at clinic level |
|  |  | Forcing people to test can lead to disengagement |
| Health education, rules and responsibility | Patients rely on HCWs for health education |  |
|  | HIV-positive people are given and extensive list of adherence and 'positive living guidelines’ | Excellent attendance, adherence and obedience |
|  |  | Learn HIV language and support the biomedical rationale |
|  |  | Reduce sexual activity and eliminate alcohol |
|  |  | Reduce and plan pregnancies |
|  |  | Eat healthy foods |
|  |  | Do not stress |
|  |  | Embrace HIV positive status/ART and help others with HIV |
|  | Biomedical model and counselling ignores social responsibilities and lived realities | HCWs often put all the responsibility of being adherent on the patient |
|  |  | Adherence monitoring and lab results prioritised over patients’ concerns about side effects and health education |
|  |  | People want to date and have relationships and need advice not only on using condoms |
|  | Peoples responses to ART guidelines and rules | Some people trust HCWs, respond well to authority and like the guidelines |
|  |  | Being able to adhere to guidelines helps some feel good about themselves |
|  |  | Stress, guilt, shame and anxiety if they are unable to abide |
|  | Labelling and punishment | Offering advice vs. delivering a reprimand |
|  |  | Inability to adhere to rules may result in being labelled |
|  |  | Labelling justifies poor treatment |
|  |  | Shame and punishment leads to disengagement and reluctance to return |
|  |  | Patients fear coming to clinic and asking stupid questions |
| Competing gender and professional norms | Inability to separate power conferred by gender vs professional role | Male providers are accustomed to positions of clear authority and social power |
|  |  | Male providers are limited by personal gender lens |
|  |  | Male patients refuse to recognise and respect clinical knowledge and authority of female providers |
|  |  | Gender inhibits provider ability to establish professional boundaries |
|  |  | Gender limits providers ability to openly discuss sex |
| **Theme 6: Poor clinic services for patients and inadequate support for health workers** | | |
| **Sub-theme** | **Codes** | **Sub-Code 1** |
| Physical space and layout of clinics | Signage, spatial and temporal problems, structures outside of the clinic are not optimal | Lack of privacy |
| It is difficult and stressful attending public ART health services | Practices in the clinic are disempowering and dehumanising | Disrespectful health workers and poor communication |
|  | Inconvenient and inflexible health services | Rigid clinic policies |
|  |  | Limited operating hours and long waiting times |
|  |  | Requirement for a treatment supporter |
|  | Navigating HIV health services is confusing and unpredictable | Clinics where staff is friendly and available confer a sense of care |
|  |  | Lack of adolescent-specific services |
|  |  | Lack of collaboration between community and HIV services |
|  |  | Lack of support groups and social activities within communities |
|  | Negative clinic experiences discourage engagement and reduce confidence in HIV care | Language barriers |
|  |  | Protocols and workload do not allow HCWs to treat everyone in the same way |
|  |  | Lost files and poor record keeping |
|  |  | Having to redo lab tests |
|  |  | Drug stockouts |
|  |  | Experiences of friends and family influences choice of clinic |
|  |  | Disregard for older people |
| It’s hard work being a health worker | HCWs do not receive adequate training on providing care for MSM | HCWs can discriminate against other HCWs if they offer quality care to MSM |
|  |  | Some HCWs fear conflict with MSM |
|  | Staff shortages and excessive work-load |  |
|  | Unable to offer solutions to patients social problems |  |
|  | Service provider burnout |  |
|  | Staff are under pressure to have good treatment outcomes |  |
| **Theme 7: The new normal requires daily drugs** | | |
| **Sub-theme** | **Codes** | **Sub-Code 1** |
| **Accepting HIV is difficult** | Initial acceptance of diagnosis is often related to perceived risk |  |
|  | An HIV diagnosis is often met with shock and disbelief |  |
|  | Denial of HIV status is a common initial response and many want to re-test |  |
|  | Coping with the diagnosis | After diagnosis people have to grapple with the possible loss of identity and future aspirations |
|  |  | Some turn to substance abuse to help them cope with the diagnosis |
|  |  | Some feel hopeless and depressed after diagnosis - |
|  |  | Some people reach a level of apathy, they don't care and wait to die |
|  |  | Accepting HIV status helps to overcome fear of judgement |
|  |  | Finding purpose and belonging through e.g. hobbies and sports helps ALHIV cope with diagnosis |
|  | Over time, most people learn to accept an HIV diagnosis | Multiple tests can help to accept diagnosis |
|  | It is hard to initiate ART if you haven’t accepted your HIV diagnosis | People who don’t feel ready to initiate ART make use of avoidance tactics: discarding ART, switching clinics, providing false information |
|  | Advancing treatments make it easier for older people to accept diagnosis |  |
|  | Despite apparent acceptance many still hope for a cure |  |
| **Time and health status may influence motivation to engage in care** | People may need time to prepare to start ART, making same day ART initiation a challenge |  |
|  | Health status markedly influences motivation to engage in care and adhere to ART | Difficult to accept ART when experiencing severe side effects |
|  |  | HIV related illness is a motivator to initiate and maintain art |
|  |  | Feeling healthy reduces motivation to initiate ART |
|  |  | Improvement of health status allow patients to live normal lives which may impair motivation to continue ART |
|  |  | Health benefits of ART lead to sustained adherence |
|  |  | Some believe that ART cures HIV |
|  |  | Concerns about long-term effects of life-long ART |
|  | Fear of death leads to questioning the value of taking ART |  |
| **People need to adapt to the medicalization of life** | Accepting the biomedical rationale helps but is not essential | Knowledge about HIV and ART helps to accept |
|  |  | Some fully accept the biomedical rationale |
|  |  | Others accept ART but maintain their own belief system |
|  |  | Although times have changed, people still believe in tradition - accepting biomedical with traditional beliefs |
|  | Patient need to accept to concept of life long treatment in order to adhere to ART | Some people have to change their jobs and lifestyle completely |
|  | A lot of changes have to made in people’s lives to incorporate ART | HIV status competing with other activities, longings |
|  |  | Want to discuss impact of HIV on future |
|  |  | Influence of alcohol on adapting to ART - influences relationship (support) and adherence |
|  |  | Changing social behaviour such as drinking alcohol |
|  | Some learn to adapt HIV/ ART guidelines to suit their needs | Some negotiate rules like drinking or having sex with multiple partners |
| **First line treatment failure** | Second and third line treatment seen as second chance, catalyst for changes and incorporating ART into life |  |
|  | Some experience regret and self-blame when reflecting on behaviour with first-line treatment (experiencing loss) |  |
| **Enabling effects of ART** | ART enables a meaningful life | Caring and providing for family |
|  |  | Being able to work |
|  |  | Being independent is important |
|  | ART can help restore a sense of masculinity | For men, ART enables having sexual relationships, physical strength to work, fulfil role as head of the household and taking care of family |
|  | Potential to reduce and resist stigma | ART can prevent unintended disclosure due to health effects |
|  |  | Some people succeed to resist stigma, disclose openly and are therefore 'free' to live a nomal life, |
|  | Psychological benefits | Reduced stress |
|  |  | Feeling better about oneself |
|  | Restores sense of belonging | early initiation of ART (when still healthy) will allow one to maintain good health, strength and energy, to work and function as a member of society |
|  | Some form a new identity | Accept a new version of self and create a new identity |
|  |  | Some generate a new advocate/activist identity |
|  | Societal acceptance of HIV | HIV becoming normal in community |
| **Theme 8: Self-efficacy, social responsibility and support helps** | | |
| **Sub-themes** | **Codes** | **Sub-code** |
| **Resilience** | People vary in how they respond to life’s challenges and have variable self-efficacy | For ALHIV taking care of one's physical, mental and emotional health is important |
|  | Social capital is a resource | Family support, time and method of disclosure (ALHIV), participating in religious practices |
|  |  | Partners’ support increases resilience |
|  |  | Networks of other patients |
|  |  | Healthcare workers |
|  | Resilience key in overcoming difficulties | self-acceptance, acknowledgement of self-worth, ambitions and plans for future, helping others, perseverance |
|  | Feeling hopeful can help with engagement | Desire to be healthy |
| **Familial responsibilities motivate people to stay on ART** | Protecting and caring for children and family is a motivator | Motivation to have an HIV negative child |
|  |  | Having a family or children to support |
|  |  | Frequent contact with health services due to sick child can help people stay in care or re-engage |
| **Supportive environments and structures help HIV positive people cope with HIV and ART** | Support helps people cope with HIV, rebuild self-esteem and offsets the effects of stigma |  |
|  | Some look to spirituality, allopathic healers or organized religion for support | Combining traditional and biomedical approach encourage patients to use ART |
|  |  | Prefer Traditional health practitioners trained in recognising HIV/AIDS symptoms |
|  | Supportive health services help people cope with the diagnosis and long term treatment | HIV specific health services often create a nurturing environment |
|  |  | HIV counselling can help patients reframe HIV and cope with diagnosis |
|  |  | Adolescent-friendly clinics that offer after school clinic hours |
|  |  | Mobiles services, increased ART days, free services, improved access/patients used problem solving strategies such as transferring care/ health outcomes |
|  | Respectful and supportive healthcare workers can make a difference and creates a strengthened sense of connectedness | Patients want relationships with providers beyond physical wellbeing |
|  |  | HCWs partnering with patients (to personalise medical regimes, talking through conflicts with family members, linking patients with psycosocial counselors/support groups, facilitating transfers to other facilities that better fit their patients' needs) |
|  |  | Support for transport and food |
|  |  | Go beyond what is expected |
|  |  | Speaks local language |
|  |  | Build rapport – reassure patients, calm the mind, explain illness in simple terms, maintain confidentiality, non-discriminatory |
|  |  | Friendly and welcoming – not act superior |
|  |  | Using connections to find drugs during drug stock-outs |
|  |  | Competence instills trust |
|  | Community health workers and community organizations offer a variety of support services and often help patients re-engage in care |  |
|  | Financial support and food parcels can help alleviate economic challenges however dependency may have unexpectedly negative results |  |
| Support from peers | Importance of peer communication and visibility of people living openly with HIV as role models | People living with disabilities role models to come out publicly and educate others |
|  | HIV positive people often support each other to cope with diagnosis and treatment | Adolescent support groups help |
|  | Therapeutic citizenship | Becoming part of the HIV community, some are passive and some are active |
|  |  | Support groups come up with innovative ways to support each other |
|  | Integrating peer counselling into support groups | Support to stop drinking for key AOD populations |
|  |  | Empower MSM through peer navigators |
| **Support from partners** | Partners can provide instrumental, informational and emotional support | Seroconcordant couples attend clinic appointments together, take ART together and remind each other |
|  |  | Serodiscordant couples encourage partners to follow rules and guidelines |
|  |  | Support from partner to manage alcohol |
|  |  | Poor relationship functioning hinders support |
|  |  | Women encourage healthy behaviour |
| **Family, friends or teachers can have a substantial impact** | Children supporting parents | Adult children providing material resources (food shelter etc) |
|  |  | Children providing emotional support |
|  |  | Helping parents to self-manage |
|  | Friends | Help to collect drugs |
| **All forms of support; practical, emotional, or financial, can help HIV positive people cope with HIV and ART** | Livelihoods, cultural practices and relationship networks |  |
| **Theme 9: The tipping point** | | |
| **Sub-theme** | **Codes** | **Sub-Code 1** |
| Complex interplay between several external and personal influences drives engagement decisions | Cyclical relationship between adherence and relationship dynamics in sero-concordant couples |  |
|  | Patients’ knowledge and understanding of HIV and ART changes over time |  |
|  | Second-line treatment may facilitate re-engagement (seen as second chance) or disengagement due to increased burden of care |  |
|  | Constant weighing up of benefits and risks of treatment-taking and status exposure |  |
|  | Relationships with HCWs | Some are scared to re-engage due to fear, blame and shame |
|  |  | Pre-established positive relationships with HCWs can help sustain engagement |
|  | Readiness to start ART |  |
|  | Resources, health status, social hierarchies, family responsibilities and clientship influence engagement and participation in care |  |
| Continuum from illness to engaging in care and medication to feeling healthy | Experiences of hope |  |
|  | Physical health declining or getting better |  |
|  | For some people one initial transitory event leads to a missed visit and other factors result in sustained disengagement |  |
|  | For some there is final event often represents a tipping point and not full picture | Getting sick is often then final factor which drive people to engage or re-engage in care |
|  |  | Poor clinic services are cited as the final cause for disengagement for many |
