# Supplementary material for: Testing for saturation in qualitative evidence syntheses: An update of HIV adherence in Africa
Source: PLoS One. 2021 Oct 19;16(10):e0258352. doi: 10.1371/journal.pone.0258352 (PMC8525762; doi:10.1371/journal.pone.0258352)
Supplement: S1 Checklist — (DOCX) [file pone.0258352.s004.docx]

**ENTREQ STATEMENT – CHECKLIST**

Enhancing transparency in reporting the synthesis of qualitative research: the ENTREQ statement: Tong et al. BMC Medical Research Methodology 2012, 12:181 (<http://www.biomedcentral.com/1471-2288/12/181> )

| **No** | **Aim** | **State the research question the synthesis addresses** | **Addressed** | **Support from manuscript** |
| --- | --- | --- | --- | --- |
| 1 | Synthesis methodology | Identify the synthesis methodology or theoretical framework which underpins the synthesis, and describe the rationale for choice of methodology (e.g. meta-ethnography, thematic synthesis, critical interpretive synthesis, grounded theory synthesis, realist synthesis, meta-aggregation, meta-study, framework synthesis). | Yes, in our original review,^[[1]](#footnote-1)^ we used a grounded theory approach and thematic synthesis to identify themes and subthemes; and developed a theoretical model. In this update of the qualitative evidence synthesis, we used our theoretical model as the framework to code new studies and to assess how they influenced original findings. | “The two authors (AR and LH) independently read and coded included studies. We coded studies using the existing list of codes and adding to the list of codes if new codes emerged. For each new code we noted whether it:   1. confirmed the findings of our original review, 2. extended the existing themes, 3. deepened or enriched our understanding of existing themes by providing illustrative examples or rich descriptions of experiences, or 4. refuted the findings of our original review.   When we developed these criteria, we also intended to examine if the studies crystallised our understanding of the themes, referring to instances where we could see the connections within and between themes more clearly. We learnt as we conducted the review that the data helped crystallise at a higher level, in terms of understanding our theory.  The two authors discussed and agreed on coding and findings for each study. The whole team met regularly to discuss new codes, and to identify any new themes or sub-themes. Through this iterative process we also discussed the impact of new codes on our proposed theory and conceptual model.” |
| 2 | Approach to searching | Indicate whether the search was pre-planned (comprehensive search strategies to seek all available studies) or iterative (to seek all available concepts until they theoretical saturation is achieved). | Yes, a pre-planned comprehensive search was conducted as per the original review | “We used the same search strategy as per the first version of this review [2] for studies published from December 2016 (the date of the last search of the original review) and 8 November 2019.” |
| 3 | Inclusion criteria | Specify the inclusion/exclusion criteria (e.g. in terms of population, language, year limits, type of publication, study type). | Yes, inclusion and exclusion criteria were the same as per the original review | “We identified new studies that met the same eligibility criteria as in the first version of this review [2]. We considered qualitative studies conducted in low-and middle-income countries (LMICs) that explored perspectives, perceptions and experiences of people living with HIV (PLHIV), caregivers and healthcare providers on linkage to and retention in HIV care, as well as adherence to ART.” |
| 4 | Data sources | Describe the information sources used (e.g. electronic databases (MEDLINE, EMBASE, CINAHL, psycINFO, Econlit), grey literature databases (digital thesis, policy reports), relevant organisational websites, experts, information specialists, generic web searches (Google Scholar) hand searching, reference lists) and when the searches conducted; provide the rationale for using the data sources. | Yes, data sources are detailed in the original review. We did not repeat these in the update. | In the original review, we state that: “We searched Medline, Embase, CINAHL, PsychInfo, LILACS, Global Health Library (date of last search 4 December 2016) and the Proquest Dissertation and Thesis database (9 December 2016). We limited our search to studies published from 1 January 2013 to include the most recent literature. Our search strategy included terms related to HIV, retention in care, adherence, linkage, LMICs and qualitative data collection and analysis methods.” |
| 5 | Electronic Search strategy | Describe the literature search (e.g. provide electronic search strategies with population terms, clinical or health topic terms, experiential or social phenomena related terms, filters for qualitative research, and search limits). | Yes, search strategies are available in the appendix of the original review. | The full search strategies for each database are reported in S2 Appendix of the original review. |
| 6 | Study screening methods | Describe the process of study screening and sifting (e.g. title, abstract and full text review, number of independent reviewers who screened studies). | Yes, study screening methods are detailed | “Two authors (AR and LH) took the same hands-on role they held in the original review and were familiar with the meaning and operational aspects of inclusion criteria and existing codes. They independently screened titles and abstracts of the search output and retrieved and independently screened full texts of potentially relevant studies.” |
| 7 | Study characteristics | Present the characteristics of the included studies (e.g. year of publication, country, population, number of participants, data collection, methodology, analysis, research questions). | Yes, a detailed table of included rich studies is available as S1 Table and we summarise the characteristics of rich studies in the text | “The 82 rich studies [9-90] were conducted in South Africa (n=20), Uganda (n=12), Tanzania (n=3), Zambia (n=6), Kenya (n=9), Malawi (n=4), Eswatini (n=3), Zimbabwe (n=1), Côte d’Ivoire (n=3), Mali (n=1), Democratic Republic of Congo (n=1), Nigeria (n=1), Ethiopia (n=1), Rwanda (n=1), Ghana (n=1). One study was conducted in Kenya, Malawi and Mozambique, one in Malawi and Zimbabwe, and one in Ghana, Uganda and Zambia. One study was conducted in the UK, Ireland, USA and Uganda, but we only considered data relevant to Uganda for this update. The ‘Bottlenecks study’ is a multi-country study [91] conducted in Malawi, Uganda, Tanzania, Kenya, Zimbabwe and South Africa and is reported in nine included papers.  Studies evaluated factors influencing linkage, retention in care and adherence to ART among adult PLHIV (n=32), adolescents or children living with HIV (ALHIV) (n=14), men (n=8), women (n=2), pregnant or postpartum women (n=9), sero-concordant or sero-discordant couples (n=7), men who have sex with men (MSM) (n=3), alcohol and other drug users (n=2), older PLHIV (n=2), people living with disabilities and HIV (n=1) and migrants (n=1). Poor papers (n=146) did not include any additional subgroup of participants not covered in the rich papers and therefore did not contribute to the analysis. Rich studies used a variety of data collection methods, with semi-structured, in-depth interviews and focus group discussions being the most common methods. Study participants were mostly HIV positive, but also included caregivers of HIV positive children or adolescents, healthcare providers, traditional healers and some HIV negative participants. S1 Table contains a summary of the characteristics of all ‘rich’ papers.” |
| 8 | Study selection results | Identify the number of studies screened and provide reasons for study exclusion (e,g, for comprehensive searching, provide numbers of studies screened and reasons for exclusion indicated in a figure/flowchart; for iterative searching describe reasons for study exclusion and inclusion based on modifications t the research question and/or contribution to theory development). | Yes, Figure 1 represents the PRISMA flow diagram that includes reasons for excluding papers | “We identified 301 studies meeting the inclusion criteria, after screening 3895 titles and abstracts and examining 352 full texts. Seventy-three studies were not conducted in Africa and thus not included in the sample. We assessed 82 papers as being rich and included them in the analysis (Figure 1). We did not sample any poor papers.” |
| 9 | Rationale for appraisal | Describe the rationale and approach used to appraise the included studies or selected findings (e.g. assessment of conduct (validity and robustness), assessment of reporting (transparency), assessment of content and utility of the findings). | We assessed studies as being rich or poor in conceptual detail. We only sampled rich studies for the analysis. | “From eligible studies, we purposively selected all studies conducted in Africa, and two authors (AR and LH) independently assessed them as being ’rich’ or ‘poor’, referring to higher or lower levels of conceptual detail within the study [8].” |
| 10 | Appraisal items | State the tools, frameworks and criteria used to appraise the studies or selected findings (e.g. Existing tools: CASP, QARI, COREQ, Mays and Pope [25]; reviewer developed tools; describe the domains assessed: research team, study design, data analysis and interpretations, reporting). | We describe the questions that guided our assessment of ‘rich’ and ‘poor’ studies. | “We defined ‘rich’ studies “according to the depth of analysis reflected in the primary study authors interpretation of findings, including any of the following features: 1) the extent to which the authors transformed or analysed their findings (beyond lists of barriers and facilitators), 2) insight into participants perspectives was demonstrated, 3) richness and complexity had been portrayed (variation explained, meanings illuminated), and 4) theoretical or conceptual development”, as per the original review [2].” |
| 11 | Appraisal process | Indicate whether the appraisal was conducted independently by more than one reviewer and if consensus was required. | Yes, two authors independently assessed papers as being ‘rich’ or ‘poor’ | ““From eligible studies, we purposively selected all studies conducted in Africa, and two authors (AR and LH) independently assessed them as being ’rich’ or ‘poor’, referring to higher or lower levels of conceptual detail within the study [8].” |
| 12 | Appraisal results | Present results of the quality assessment and indicate which articles, if any, were weighted/excluded based on the assessment and give the rationale. | We only included rich studies in the analysis. |  |
| 13 | Data extraction | Indicate which sections of the primary studies were analysed and how were the data extracted from the primary studies? (e.g. all text under the headings “results /conclusions” were extracted electronically and entered into a computer software). | Yes, we extracted authors results and conclusions | “The two authors (AR and LH) independently read and coded included studies. We coded studies using the existing list of codes and adding to the list of codes if new codes emerged” |
| 14 | Software | State the computer software used, if any. | Yes, detailed | “They independently screened titles and abstracts of the search output using Covidence software [8] and retrieved and independently screened full texts of potentially relevant studies.” |
| 15 | Number of reviewers | Identify who was involved in coding and analysis. | Yes, Identified | ““The two authors (AR and LH) independently read and coded included studies.” |
| 16 | Coding | Describe the process for coding of data (e.g. line by line coding to search for concepts). | Yes, described | “The two authors (AR and LH) independently read and coded included studies. We coded studies using the existing list of codes and adding to the list of codes if new codes emerged. For each new code we noted whether it:   1. confirmed the findings of our original review, 2. extended the existing themes, 3. deepened or enriched our understanding of existing themes by providing illustrative examples or rich descriptions of experiences, or 4. refuted the findings of our original review.” |
| 17 | Study comparison | Describe how were comparisons made within and across studies (e.g. subsequent studies were coded into pre-existing concepts, and new concepts were created when deemed necessary). | Yes, detailed | “The two authors discussed and agreed on coding and findings for each study. The whole team met regularly to discuss new codes, and to identify any new themes or sub-themes. Through this iterative process we also discussed the impact of new codes on our proposed theory and conceptual model.” |
| 18 | Derivation of themes | Explain whether the process of deriving the themes or constructs was inductive or deductive. | Yes, we used our theoretical model developed in the original review as the framework to code new studies and to assess how they influenced original findings. | “The two authors (AR and LH) independently read and coded included studies. We coded studies using the existing list of codes and adding to the list of codes if new codes emerged. For each new code we noted whether it:   1. confirmed the findings of our original review, 2. extended the existing themes, 3. deepened or enriched our understanding of existing themes by providing illustrative examples or rich descriptions of experiences, or 4. refuted the findings of our original review.”   “When we developed these criteria, we also intended to examine if the studies crystallised our understanding of the themes, referring to instances where we could see the connections within and between themes more clearly. We learnt as we conducted the review that the data helped crystallise at a higher level, in terms of understanding our theory. The two authors discussed and agreed on coding and findings for each study. The whole team met regularly to discuss new codes, and to identify any new themes or sub-themes. Through this iterative process we also discussed the impact of new codes on our proposed theory and conceptual model.” |
| 19 | Quotations | Provide quotations from the primary studies to illustrate themes/constructs, and identify whether the quotations were participant quotations of the author’s interpretation. | We did not include quotations from primary studies. Our paper represents an update of the original QES, but also a case study on data saturation | “Our objective was to update the review, and then use formal appraisal to examine the changes that updating, with three years of papers that followed, had on the findings of the original review. Our approach was to evaluate data saturation in relation to coverage against the original conceptual model; and any new, enrichment or modification of the themes in the final review” |
| 20 | Synthesis output | Present rich, compelling and useful results that go beyond a summary of the primary studies (e.g. new interpretation, models of evidence, conceptual models, analytical framework, development of a new theory or construct). | Yes, we summarise the original findings and how the new studies relate to these. We also present the same conceptual model as in the original review, as we did not identify any new themes. | “Below we summarise the original findings, and how the new studies relate to this. New codes and subthemes are listed in S3 Table.”  “No new themes emerged from the updated analysis. Within the author team, we discussed changing the name of Theme 7 and splitting Theme 8 into two themes, but after careful consideration, we concluded that it made more sense to enrich and extend the original themes. The extra details, illustrative examples and in-depth descriptions of experiences contained in the new studies allowed us to paint a clearer, more colourful, and more comprehensive picture of how PHLIV understand and respond to pressures they encounter in their daily lives. We were thus able to gain greater insight into factors that influence the dynamic process of engagement, disengagement and reengagement in care, and how these factors are connected (Figure 2). In hindsight, we recognise Theme 9, ‘The tipping point’, as a crystallisation of everything described in Themes 1-8.” |

1. Eshun-Wilson, I., A. Rohwer, L. Hendricks, S. Oliver and P. Garner (2019). "Being HIV positive and staying on antiretroviral therapy in Africa: A qualitative systematic review and theoretical model." PLoS One **14**(1): e0210408. [↑](#footnote-ref-1)
