# Supplementary material for: Testing for saturation in qualitative evidence syntheses: An update of HIV adherence in Africa
Source: PLoS One. 2021 Oct 19;16(10):e0258352. doi: 10.1371/journal.pone.0258352 (PMC8525762; doi:10.1371/journal.pone.0258352)
Supplement: Attachment — Submitted filename: responses to ref comments_9 September.docx [file pone.0258352.s005.docx]

**Paper with PLOS ONE**

Title: Testing for saturation in qualitative evidence syntheses: An update of HIV adherence in Africa

**REVIEWER 1**

Thank you for a really interesting paper. Below are some comments which I feel could strengthen the content. This is an important contribution to a rapidly evolving QES field and addresses two important questions of saturation and when to update a review.

| **REVIEWER 1 - COMMENTS** | **AUTHORS RESPONSE** |
| --- | --- |
| **Abstract:** |  |
| In the paper you talk about a certain type of richness (conceptual), can this be added to the abstract to clarify. | We have revised the sentence as follows:  “We assessed studies for conceptual detail, categorised as ‘rich’ or ‘sparse’, coding the rich studies.” |
| **Introduction:** |  |
| Can the authors provide more of a definition of what they mean by rich (thick) and poor. In the introduction thick is used as a synonym for rich but can have a different interpretation when linked to thick description. I suggest changing line 53 to rich. | In the original review, we used the terms ‘thick’ and ‘thin’ to describe the depth of the analysis in included papers. However, for this updated review, we changed this to ‘rich’ and ‘sparse’, as these terms are used when referring to conceptual detail, while ‘thick’ and ‘thin’ describe contextual detail (as per guidance from the new Chapter on Qualitative Evidence in the Cochrane Handbook). We believe that conceptual detail is more appropriate and relevant when examining saturation and have now explained this in the introduction. |
| There is a debate in the social science community about whether saturation is reachable in primary research. See, for example:  Braun 2019  ([https://www.tandfonline.com/doi/full/10.1080/2159676X.2019.1704846?casa_token= yW3J1lQL49AAAAAA%3AtX3ULeoVVftcerWwaF2kmrsBv8d7F6B7Vt0q24u WoWZFszfaKYL10iqxOqCKVBJ9ojnj6CMzF9UP2e0](https://www.tandfonline.com/doi/full/10.1080/2159676X.2019.1704846?casa_token=yW3J1lQL49AAAAAA%3AtX3ULeoVVftcerWwaF2kmrsBv8d7F6B7Vt0q24uWoWZFszfaKYL10iqxOqCKVBJ9ojnj6CMzF9UP2e0))  Low 2019 <https://www.tandfonline.com/doi/full/10.1080/00380237.2018.1544514?src=recsys>)  Sebele-Mpofu 2020 ([https://www.tandfonline.com/doi/full/10.1080/23311886.2020.1838706](https://protect-eu.mimecast.com/s/KfIpCLvOzUPk2Xyt2sVt_?domain=eur01.safelinks.protection.outlook.com))  I think this same debate can apply to secondary data analysis like in a QES. I suggest the authors mention this debate and state in the reflexivity section that they believe data saturation is achievable (or not). | Thank you for suggesting these papers, we have used Sebele-Mpofu in drawing our conclusions.  In addition, to address the point about the reflexivity, we have added the following to the Reflexivity section: “All authors view data saturation in primary research as a point in data collection where additional data does not add any new insights [5] and believe that this is achievable. We were unsure whether the same principle applies to QES, which is why we undertook this study.” |
| Line 76- The authors write that QES are adopting methods of quantitative research synthesis- I don’t think this is completely correct. We are adopting some similar approaches in relation to the steps in a SR but the methods for a QES are quite different to a quantitative SR as we are much more flexible and are not necessarily trying to identify all existing literature on a topic. I suggest rephrasing. | We agree and have changed this to: “We note that researchers are applying “updating” principles of quantitative research synthesis to QES.” |
| **Material and methods** |  |
| The authors discuss studies from other LMIC settings outside of Africa, but it is unclear why these were searched for when the title is about Africa. I suggest that reference to these studies can be removed or needs to be clarified. | In the original review, we set out to assess adherence in all LMICs. However, we found very little evidence from countries outside of Africa and thus only sampled studies from Africa. As we are looking at saturation, we used the same methods as per the original review and hence studies from LMICs were eligible, but we only sampled studies from Africa.  In the methods section, we report: “From eligible studies, we purposively selected all studies conducted in Africa”.  In the PRISMA flow diagram, we report the number of articles that were eligible for inclusion but not sampled, as well as the reasons for not sampling these papers. |
| I would like more detail around how the authors decided if a study was rich or poor. They refer to levels of conceptual data and depth of analysis but not to thickness or richness of the data in the article (more traditional definitions of thick description). I do not feel that with the level of detail provided another author team could go out and make the same assessments for the included studies. Did they use a checklist?  Only discussion? What was the cut-off point? Is it a yes or no or a sliding scale? Could they provide examples in a supplementary document of the decision process for a rich and poor study? I think this would be useful for future author teams to use the same approach. | See comment above.  We used four features of ‘rich’ studies to guide the decision on whether a paper was categorised as ‘rich’ or ‘sparse’. Two authors independently read papers and assessed richness. We then discussed papers and decided on the final category by consensus. Although we had intended to use a checklist with scores, we did not have good consensus when piloting this approach.  We have now added a more detailed description of this process to the methods section. |
| Line 105- The authors write that they sampled poor studies…. But in the next section they write that they did not. Could 105 be changed to “we would have sampled…” | Changes made as suggested. |
| Reflexivity See comment above on adding their standpoints on data saturation to the reflexivity section | See previous response on reflexivity. |
| **Results** |  |
| Line 140- Again I think reference to non-African studies can be removed. | We prefer to keep this as is. |
| Line 145- Can you please reorder the countries alphabetically or by N | Countries reordered by N. |
| Line 160- Were all the included studies single methods studies or did any use methods triangulation? Was this considered in your rich vs poor evaluation? | We describe data collection methods in the table of included studies (S1 Table). 39 studies used more than one method to collect data – we have added this to the results section. |
| Line 209- Do you mean death sentence rather than death threat. A death threat seems odd here. | Changed as suggested. |
| Line 253- Is there a word missing from the end of theme 5 (system?) | Indeed - thanks for picking this up. |
| In general, throughout the results section I feel there is a need for qualifying language when it comes to participants. Often it is written as if all the participants were expressing the same point of view. I.e. line 266 Male HCWs are accustomed to…. This is a very broad statement and it would be difficult to see if all male HCWs felt this way. I feel it should be tempered by some, many, those male HCWs included in the sampled studies etc. | We checked the results section and made changes as suggested. |
| **Discussion** |  |
| In general, the authors argue that they have reached data saturation at the thematic level. However, they highlight that the new studies provided new insight and nuance to existing themes (extended, enriched). I would argue that this is a finding and that perhaps data saturation was not reached. I would like to seem some reflection on if they feel this new data was important enough that the review should be updated even though no new themes were added. Would the new extended and enriched data have implications for policy or practice especially around the new subgroups that were identified? | We think there was thematic saturation. New studies provided some additional richness. Any new study will add data, but the question is whether it will make a material difference to the theory. We have already offered these examples in the results section where we consider whether the new studies confirm, enrich, extend or refute our themes.  This clearly shows that most studies confirmed our findings within the themes. None of the studies refuted our theory.  We would therefore argue that this does indicate that saturation at thematic level was reached, which is why we did not change our conceptual model depicting the theory. |
| The authors provide useful information for QES groups contemplating updating a review. | Thanks. |
| I appreciate the reference to CERQual as I think this could be a useful measure. Did their findings move from low to moderate or high confidence with addition of the new studies? There has been one review to use CERQual on theoretical findings that will be published shortly (Cooper vaccination review with EPOC). | In our original review, a theme would not make the final cut unless we had high level of certainty in our interpretation.  Our work shows that another 82 studies indeed confirmed the findings in the previous review, and in some areas enriched this. This is hardly surprising, but what is important is that it did not fundamentally impact on the findings, nor or make things “more certain”.  We added the following to the discussion:  “We applied the same principle of presenting rich findings to the original and updated synthesis, and only included findings if we had high confidence in them. We therefore had no opportunity to consider whether confidence in particular themes was increased, as might happen if studies were conceptually sparse rather than conceptually rich as we would not include them if there was doubt as to their validity. The new studies included in this update confirmed our confidence in our original theory. We did not use the GRADE-CERQual approach which is designed for assessing individual descriptive findings, as it is not currently known how well it performs when assessing higher order analytical findings and theory building [3, 95]. We are exploring ways to use the GRADE-CERQual approach on this dataset, and this is a separate piece of work in progress.” |
| **Conclusion** |  |
| Line 427- Yes, no new themes were found but existing themes were enriched and extended in a very useful and interesting way and this is worth mentioning. | We have amended the conclusion. |
| Other questions to consider but do not need to be added to the article:  Do the methods used in the included studies impact your certainty of reaching saturation? For example, if there are limited observation studies or a number of studies that only used a single method for data collection does this impact on a QES saturation? | Thanks for these questions. As per the previous responses, we only included studies with rich data in the analysis. |

**REVIEWER 2**

I was extremely impressed by this article which is generally well-written and contributes well to the emerging evidence base on qualitative evidence synthesis updates. I had a few very specific points about phrasing and a few important points of accuracy and precision.
My main reservation is the quality of the Discussion – the authors touch on important issues but fail to engage sufficiently with the existing methodological literature. There is already a small body of evidence about sampling of qualitative evidence and also about conducting qualitative sensitivity analysis for quality as well as some recent work on richness and on relevance and on context. While I am not expecting the authors to engage equally with all of these methodological literatures the contribution of their article would be confirmed, extended and enhanced by pursuing at least one or two of these issues to take the contribution beyond an update case study.

| **REVIEWER 2 - COMMENTS** | **AUTHORS RESPONSE** |
| --- | --- |
| **REVIEWER 2** | |
| **Abstract:** |  |
| rationale, triggers, and methods for updating is less clear cut. “is” should be “are” | Changed as suggested. |
| “301 studies met the inclusion criteria. Rich findings from Africa were available in 82 studies; 146 studies were poor” This makes 228 studies - what happened to the remainder? | The rest were studies that were conducted in LMICs outside of Africa. We only sampled studies conducted in Africa. |
| “careful appraisal of the new literature” – maybe “appraisal” is not a helpful term here because of its stronger technical association with quality assessment. Maybe “evaluation”? | Changed as suggested. |
| **Introduction:** |  |
| “when new data just repeats was has already been found in previous data” – “what” not “was” | Changed as suggested. |
| “As researchers are now adopting methods of quantitative research synthesis for QES and are discussing updating QES [5-7]” – This sentence is misleading – the point being made seems to be only relating to updating and I don’t see anything in the three cited references that demonstrate “methods of quantitative research synthesis” being used in QES. Something like: “As the increasing prevalence of QES is leading to an imperative to update QES that follows that previously encountered within quantitative research synthesis [5-7]” or similar. | See similar comment from Reviewer 1. We changed this to: “We note that researchers are applying “updating” principles of quantitative research synthesis to QES.” |
| In the Abstract you refer to “confirmed, extended, enriched, or refuted”. In the Introduction it is: “new, enrichment or modification” – it might be helpful to use a common terminology. | We agree and have amended the sentence accordingly. |
| “We defined ‘rich’ studies “according to the depth of analysis reflected in the primary study authors interpretation of findings “” It might be helpful to acknowledge different approaches to operationalising “richness” of which the most developed is Ames, H., Glenton, C. & Lewin, S. Purposive sampling in a qualitative evidence synthesis: a worked example from a synthesis on parental perceptions of vaccination communication. BMC Med Res Methodol 19, 26 (2019). [https://doi.org/10.1186/s12874-019-0665-4](https://protect-eu.mimecast.com/s/pNIrCNYgBSNZp9DTLlXaJ?domain=eur01.safelinks.protection.outlook.com) | See response to Reviewer 1. |
| “any additional subgroups not covered” – it is difficult at this point in the manuscript to understand what these “subgroups” might be – perhaps an example here e.g. are they population groups? | We have changed this to “population groups” to clarify. |
| “We report the number of studies confirming our original findings, extending existing themes, and enriching our understanding of existing themes” What happened to “refuting”? | We have amended the sentence to include “refuting” |
| “We did not sample any poor papers.” State number of poor papers here “We did not sample any of the X poor papers”. (NB. We tend to use “sparse” instead of poor because it is unambiguously not about quality but I appreciate this may be personal preference). | Thanks for the suggestion. We agree and have now replaced ‘poor’ with ‘sparse’. |
| “described a lot of positive effects” – “a lot of” is colloquial for a scientific paper. | We have replaced ‘a lot of’ with ‘various’ |
| “None of the studies refuted our theory, despite careful consideration within the team of possible adjustments being made.” I think that more could be made of this statement – i.e. what you were looking for and why you might not have found it. | None of the studies contradicted our theory that there are multiple factors influence the dynamic process of engagement, disengagement and reengagement in care. Within specific themes, new data either confirmed, extended or enriched our understanding of the theme. The findings from new studies were thus very similar to the ones included in the original review. As reported, there were no major policy changes within the timeframe of these studies, and it is therefore not unexpected that new data did not refute our theory. Our findings are based on a substantive literature base. There were no red flags that made us doubt our theory. We described how we considered changing and/or splitting themes in the discussion. |
| I particularly liked your confirmed, extended enhanced descriptors as used when assessing the value of each revisited theme.  Methodologically there might have been a justification for a framework synthesis approach given the fact that you wanted to revisit the original analysis and see what an update might add. You could at least consider this methodological option within the article. | Thank you so did we.  We recognise the similarities between the methods applied and framework synthesis. However, our starting framework was not ‘constructed’ and was more than a ‘best fit’ in that the scope was identical so differs from the two main approaches to framework synthesis. We think our description of starting with a framework to test saturation is clear, and any allusion to framework synthesis may confuse readers. |
| **Discussion:** |  |
| this is disappointing as it identifies potential issues but does not relate to published literature– issues that could be considered include absence of refutational evidence (compare experience in meta-ethnography), differential definitions of richness (Ames paper above), the fact that poor quality studies [“these poor studies may fulfill other more immediate purposes such as informing specific programmes”] have been shown to add little conceptually to analyses but may add new contexts (Carroll & Booth). Also that new studies may automatically add new contexts temporally speaking (changing attitudes, contextual factors eg Covid) and that you may only know whether the new studies are worth including by doing this – i.e. you can’t predict whether an update will be worth doing in advance. | Thanks for these helpful comments and we have amended this section to take in these points. |
| “In our previous review we had sampled thin studies” – the concept of “thin” studies is new to this paper – before you used “poor” Are they the same or different? | We used the terms ‘thick’ and ‘thin’ in the original review, but have now changed this to ‘rich’ and ‘sparse’. |
| “It raises the question whether updating a QES which already has a substantive literature base should be done without very considerable thought if this is a sensible use of resources.” (“about whether” not “if”) | Changed as suggested. |
| This is a very simplistic and quantitative conclusion which is disappointingly at odds with the rest of the paper. For me, instead, this raises the question “what constitutes a “substantive literature base”?” It surely can’t just mean number of studies? So what else could it mean? And what might a review team do to decide whether theirs is “substantive” or not? Also might the type of analysis be a factor e.g. meta-ethnography versus thematic synthesis?  I also wonder whether the Discussion would be enriched by some consideration of what saturation means in the context of primary research. | We think that if there is a good theory, with lots of rich literature, there needs to be a really good reason to redo this.  We still think that if you are confident of the theory and there is no obvious policy shift then there is not a good a priori reason to automatically conduct an update.  We have amended the conclusion. |
| “good baseline understanding of the theory,” – Use of “baseline understanding of the theory” is a strange choice of expression – theories are qualitative and therefore don’t have a baseline. We would typically refer to “a good grounding in the theory”. | Changed as suggested. |
| “then using the existing team is economical and sensible.” – economical, maybe but an alternative view about “sensible” is that for an interpretive work introducing new perspectives could enhance the analysis – looking at differences not similarities. Covered in Booth “disconfirming case” article. | We have added the following sentence and have cited the paper as suggested:  “However, we acknowledge that adding new authors to the team could have enhanced our analysis by introducing different perspectives, particularly on differences in the data.” |
| “CERQual tool” – the approved designation is “GRADE-CERQual approach”. It is not described as a “tool” – it includes multiple processes. Also its purpose is not “formally assessing quality of themes” it is “for assessing how much confidence to place in findings from systematic reviews of qualitative research (or qualitative evidence syntheses)”. | Changed as suggested. |
| Also the statement “designed for assessing individual descriptive findings rather than higher order analytical findings, and theory building” seems to imply that GRADE-CERQual cannot be used for the latter. A more accurate statement is that “and it is not currently known how well it performs when assessing higher order analytical findings, and theory building”. Also the reference should be to a paper in the newer GRADE-CERQual series not to the original PLOS article. | Changes to text and references made as suggested. |
| “Our updated QES of adherence to ART strengthened our theory” Again I think that you should utilise your previous descriptors of “confirmed, extended, and enriched” unless that is you are invoking the “crystalise” analogy of earlier which would imply a higher level of contribution. | We changed this to: “Our updated QES of adherence to ART confirmed our confidence in the theory proposed in the original version of the review, and at times also extended and enriched existing themes.” |
